# TMPA-HC: a two-stage heterogeneous multi-population algorithm with cooperative search for high-dimensional feature selection

**DOI:** 10.1038/s41598-026-48133-x

**Published:** 2026-04-12

**Authors:** Aolin Chen, Pengfei Pan, Ning Quan, Bo Zhou

**Affiliations:** 1 Faculty of Mechatronic Engineering, Xuzhou College of Industrial Technology, Xuzhou, 221140 China; 2 Department of Applied Mathematics, University of Washington, Seattle, WA 98195 USA

**Keywords:** High-dimensional Feature Selection, Multi-population optimization, Cooperative search, Fisher filter, Lévy flight, Computational biology and bioinformatics, Mathematics and computing

## Abstract

Feature selection is a fundamental yet challenging task in machine learning, particularly in high-dimensional settings. Although swarm intelligence and evolutionary computation methods, including ant colony optimization and grey wolf optimizer, have shown promising performance in feature selection, they still face two major limitations in high-dimensional spaces. First, the selected feature subsets often contain considerable redundancy, which negatively impacts the performance of classifiers. Second, the computational cost increases rapidly with dimensionality, leading to unsatisfactory efficiency in practical applications. In response to the above challenges, this study introduces TMPA-HC, a two-stage heterogeneous multi-population framework that employs cooperative search for high-dimensional feature selection. The proposed approach adopts a two-stage framework that integrates an initial Fisher-score-based filtering stage with a subsequent wrapper-based heterogeneous multi-population optimization stage. In the second stage, the population is divided into multiple subpopulations with distinct search roles, enabling structured exploration-exploitation behaviors. To facilitate effective collaboration, TMPA-HC incorporates several cooperative mechanisms, including elite cross-population hybridization, cyclic information transfer, and subpopulation reorganization. In addition, a success-rate–driven adaptive control strategy dynamically adjusts the search intensity of each subpopulation, while lightweight elite-guided local search and stagnation-aware restart mechanisms enhance convergence stability and robustness. Comprehensive experiments on multiple high-dimensional benchmark datasets show that TMPA-HC achieves competitive feature selection performance with consistent convergence, demonstrating its effectiveness and stability in handling high-dimensional data.

## Introduction

Modern data-intensive applications across domains such as biomedical analysis^1^, text mining^2^, and remote sensing^3^ routinely generate extremely high-dimensional datasets^4^. Although such high-throughput data offer rich opportunities for knowledge discovery, they also contain substantial noise, redundancy, and irrelevant variables^5,6^. These factors inflate computational costs, increase model complexity, hinder interpretability, and exacerbate the risk of overfitting^7,8^. Consequently, developing effective mechanisms to cope with high-dimensional data has become a critical requirement in contemporary machine learning.

To alleviate the challenges associated with high dimensionality, dimensionality reduction techniques are generally categorized into feature extraction^9–11^ and feature selection (FS)^12–14^. Feature extraction methods, including Linear Discriminant Analysis (LDA)^15^ and Principal Component Analysis (PCA)^16,17^, generate concise representations by mapping samples into a lower-dimensional latent space. However, such transformations inevitably distort the semantic meaning of original features^18^, which limits their usefulness in applications where interpretability is essential^19^. In contrast, FS preserves the original feature space while identifying an informative subset of variables, thereby enhancing interpretability and reducing computational burden^20,21^. These characteristics make FS particularly suitable for high-dimensional tasks that involve noisy or redundant attributes^22,23^.

Despite its advantages, FS remains challenging in high-dimensional environments^24^. First, the abundance of irrelevant or redundant features drastically enlarges the search space, making the identification of high-quality subsets difficult and prone to stagnation^25^. Second, the computational burden increases sharply with dimensionality, leading to slow convergence and poor scalability for many existing FS methods^26,27^. Accordingly, designing FS approaches that remain efficient, accurate, and robust in ultra-high-dimensional scenarios is an active and demanding research problem^28^.

To address FS from an optimization perspective, population-based optimization and metaheuristic search algorithms have been proposed. Swarm intelligence methods such as ant colony optimization (ACO)^29,30^, differential evolution (DE)^31^, particle swarm optimization (PSO)^32^, and various hybrid metaheuristics^33^ have demonstrated strong global search capabilities and encouraging performance on complex, multimodal search spaces. Nevertheless, most single-population evolutionary algorithms suffer from inherent limitations, including reduced population diversity, premature convergence, and difficulties in maintaining a proper balance between exploration and exploitation. These drawbacks often result in redundant or suboptimal feature subsets, especially in large-scale search spaces.

To overcome these limitations, multi-population optimization frameworks have attracted increasing attention in recent years^34,35^. By decomposing the population into multiple interacting subpopulations, such frameworks are able to maintain diverse search trajectories, enhance robustness, and reduce the risk of premature convergence. Representative studies have explored island-model-based and distributed multi-population strategies to enhance diversity and mitigate premature convergence. For example, Abed-alguni et al.^36^ proposed the distributed grey wolf optimizer (DGWO), which organizes the population into multiple islands with migration mechanisms to improve global search performance. Similarly, island-based cuckoo search variants, such as iCSPM^37^ and its improved version iCSPM2^38^, incorporate structured subpopulation evolution and mutation strategies to strengthen exploration and convergence behavior. However, their effectiveness strongly depends on how heterogeneity among subpopulations is designed and how cooperation mechanisms are organized. While various multi-population approaches have introduced interaction mechanisms, a considerable portion of them rely on relatively fixed cooperation structures or predefined information exchange patterns, which may limit their ability to adaptively regulate search pressure and dynamically coordinate information flow during optimization^39,40^. In high-dimensional FS tasks, insufficient coordination or poorly designed cooperation strategies may lead to inefficient exploration or excessive computational overhead^41,42^. Therefore, there remains significant room for developing more structured and adaptive multi-population frameworks that can fully exploit cooperation while preserving efficiency.

Based on the above analysis, it can be observed that existing hybrid and multi-population feature selection methods still primarily rely on implicitly regulated exploration–exploitation mechanisms within homogeneous evolutionary structures. Although filter–wrapper combinations and multi-population strategies have been widely studied, their cooperation mechanisms are often static, loosely coupled, or lack adaptive structural regulation. As a result, maintaining diversity while ensuring stable convergence in extremely high-dimensional and small-sample settings remains challenging.

To address these limitations, this paper proposes a cooperative-search-driven two-stage heterogeneous multi-population algorithm, termed TMPA-HC, for high-dimensional feature selection. Unlike conventional hybrid approaches that simply combine filtering and metaheuristic optimization, TMPA-HC introduces a structurally decoupled heterogeneous search framework. Specifically, the search process is explicitly divided into three specialized subpopulations–exploration-oriented, exploitation-oriented, and balance-oriented groups–each assigned a distinct functional role. This structural decomposition enables parallel yet coordinated search dynamics rather than relying solely on parameter tuning to control exploration–exploitation balance.

In the first stage, a Fisher-score-based filter is employed as a computationally efficient dimensionality reduction mechanism to suppress evidently irrelevant or weakly discriminative features. This step reduces search complexity before entering the wrapper phase. In the second stage, a heterogeneous cooperative optimization framework is constructed, integrating role-based subpopulation specialization, cross-population hybridization, cyclic knowledge transfer, controlled reorganization, and stagnation-aware restart strategies.

More importantly, a success-rate–driven adaptive control mechanism is introduced to dynamically modulate subpopulation search intensities based on real-time evolutionary feedback. This feedback-coupled adaptive regulation enables TMPA-HC to self-adjust search pressure during different evolutionary stages, thereby improving convergence stability and robustness under high-dimensional conditions.

Therefore, the novelty of TMPA-HC lies not merely in combining existing components, but in designing a structured cooperative evolutionary architecture with explicit role differentiation, multi-level diversity maintenance, and feedback-driven adaptive regulation tailored for high-dimensional feature selection.

The main contributions of this work can be summarized as follows:

1. A structurally heterogeneous two-stage feature selection framework is developed, in which a computationally efficient Fisher-based filtering stage is followed by an explicitly role-decoupled multi-population wrapper optimization stage, enabling scalable search in ultra-high-dimensional spaces.
2. A role-based cooperative evolutionary architecture is proposed, where exploration, exploitation, and balance subpopulations operate with differentiated update mechanisms and controlled cross-subpopulation knowledge exchange.
3. A success-rate–driven adaptive regulation strategy is introduced to dynamically adjust subpopulation search intensities based on evolutionary feedback, enhancing robustness and preventing structural premature convergence.
4. A multi-level diversity preservation strategy operating at offspring-generation, subpopulation-interaction, and population-reorganization levels is constructed through heterosis-driven hybridization, subpopulation reorganization, elite-guided refinement, and stagnation-aware restart, forming an integrated cooperative search mechanism.
5. Extensive experiments on benchmark functions and multiple high-dimensional biomedical datasets demonstrate the effectiveness, stability, and competitiveness of the proposed TMPA-HC framework.

Overall, the proposed TMPA-HC provides a structured and adaptive cooperative optimization framework specifically designed to address the exploration–exploitation imbalance, diversity degradation, and scalability challenges in high-dimensional feature selection tasks.

## Related work

FS has long been acknowledged as a key approach for reducing data dimensionality by identifying informative feature subsets while retaining the essential characteristics of the original feature space^43,44^. Existing FS methods are typically divided into three categories: filter, wrapper, and hybrid approaches. Filter-based approaches assess features using statistical measures such as mutual information, correlation, or consistency, providing high computational efficiency but often resulting in suboptimal subsets due to the lack of integration with predictive models^45^. In contrast, wrapper-based approaches use an algorithm to directly assess candidate feature subsets, yielding improved predictive performance at the cost of higher computational effort due to repeated model evaluations^46,47^. Hybrid methods combine filter heuristics with wrapper refinement to balance efficiency and accuracy, making them particularly suitable for high-dimensional feature spaces^48^. This combined approach has achieved good progress in various fields^49^.

Song et al.^50^ developed a three-stage hybrid feature selection method, termed HFS-C-P, which combines correlation-based clustering with particle swarm optimization to address dimensionality challenges while reducing computational overhead in high-dimensional FS. Specifically, an initial filtering phase removes irrelevant features using symmetric uncertainty criteria; subsequently, a correlation-guided clustering mechanism aggregates redundant features to reduce the effective search space; finally, an enhanced integer PSO-based wrapper is employed to conduct global optimization and select an optimal feature subset. Evaluations conducted on 18 publicly accessible real-world datasets, with comparisons against nine competing FS approaches, demonstrated that HFS-C-P achieves competitive feature selection performance with the lowest computational complexity. Zhu et al.^51^ introduced HFSIA, a high-dimensional hybrid feature selection approach that integrates a low-cost filter with a metaheuristic search based on an artificial immune algorithm. To enhance search effectiveness and preserve population diversity efficiently, HFSIA employs a lethal mutation mechanism, an adaptive Cauchy mutation operator, and an adaptive population update strategy. When evaluated on 22 high-dimensional benchmark datasets against 23 state-of-the-art methods, HFSIA achieved computational costs comparable to five classical fast FS techniques while attaining higher average classification accuracy than 18 recent hybrid approaches, along with the highest feature reduction rate. Parhi et al.^52^ proposed SC-MBO-BLS, a hybrid gene selection and classification framework for genomic data that couples a filter with a metaheuristic wrapper. First, Kernel-based Fisher Score (K-FS) selects salient genes; next, an improvised sine–cosine–hybridized Monarch Butterfly Optimization (SC-MBO) embedded in a Broad Learning System (BLS) wrapper simultaneously searches for an optimal gene subset and trains the classifier. Evaluated on ten cancer gene-expression datasets and compared against SC-MBO-MLP, SC-MBO-ELM, SC-MBO-KELM, and 20 standard baselines, SC-MBO-BLS achieved the best overall performance across precision, MCC, sensitivity, Kappa, F-score, and specificity, with accuracies ranging from 97.2% to 100%; ANOVA confirmed the superiority of the method. Ma et al.^53^ introduced TSHFS-ACO, a two-stage hybrid ACO high-dimensional feature selection method. In the first stage, an interval strategy estimates the optimal feature subset (OFS) size, reducing search complexity and mitigating the risk of local optima. In the second stage, an enhanced ACO algorithm with a hybrid evaluation model utilizes both intrinsic feature relevance and classification performance to guide the search for the OFS. Experimental results on eleven high-dimensional public datasets demonstrate that TSHFS-ACO achieves state-of-the-art performance on most datasets while requiring less runtime than other ACO-based FS methods. Shen et al.^54^ introduced IGWO, a two-stage improved grey wolf optimizer(GWO) high-dimensional feature selection framework, which integrates a sparsity-inducing surrogate with an efficient discrete search strategy. First, an MLP with group-lasso regularization is trained to formulate an integer optimization for feature pre-selection and hidden-layer structure tuning, yielding a compressed dataset; second, the group-lasso MLP is retrained on the compressed data while IGWO solves a discrete FS problem guided by a rapid evaluation strategy to cut assessment cost. On ten gene-expression datasets, the method removed over 95.7% of features across all datasets and achieved superior test accuracy, with advantages in runtime, accuracy, and subset size becoming more pronounced as dimensionality increases. Ling et al.^55^ proposed a two-stage multi-modal, multi-objective PSO algorithm for feature selection that leverages feature importance to both prune the search space and enhance niching. First, feature importance is computed by integrating Spearman’s rank correlation and the maximal information coefficient to eliminate redundant and weakly correlated features, thereby reducing the decision space; second, a feature-importance–guided mutation is applied to the optimal particle within each niche to help dominated particles escape local optima and refine multiple equivalent subsets. Experiments on ten datasets showed that the method identifies lower-dimensional, superior equivalent feature subsets without compromising classification accuracy.

While supervised methods rely on label information, unsupervised feature selection has also garnered significant attention due to the abundance of unlabeled data. Dwivedi et al.^56^ provided a comprehensive taxonomy of unsupervised feature selection methods, highlighting their pros, cons, and implementation challenges. In this domain, swarm intelligence algorithms have shown promising results in balancing exploration and exploitation. For instance, Dwivedi et al.^57,58^ proposed hybrid feature selection approaches based on Ant Colony Optimization (ACO) that utilize clustering validity indices, such as Silhouette and Laplacian scores, to effectively escape local optima and enhance global search capabilities. Furthermore, addressing the computational bottleneck in massive datasets is crucial; Tripathi et al.^59^ introduced a Scalable Distributed Laplacian Score framework implemented on Apache Spark, demonstrating significant improvements in execution efficiency for large-scale feature selection tasks.

In addition to supervised and hybrid optimization-based feature selection approaches, semi-supervised feature selection methods have also attracted considerable attention due to the widespread availability of unlabeled data in practical applications. Sheikhpour et al.^60^ proposed HSDAFS, a sparse feature selection method based on hypergraph Laplacian-driven semi-supervised discriminant analysis. By incorporating hypergraph modeling into the semi-supervised learning framework, HSDAFS captures high-order structural relationships among labeled and unlabeled samples beyond traditional pairwise graph representations. The method formulates feature selection as a trace-ratio optimization problem and integrates mixed convex and nonconvex norm regularization to promote row sparsity in the projection matrix. This design enables joint selection of discriminative features while preserving intrinsic geometric structure. Experimental results across several benchmark datasets demonstrated that HSDAFS achieves improved discriminative capability compared to existing graph-based semi-supervised feature selection approaches. Sheikhpour et al.^61^ further introduced SCFLR, a semi-supervised feature selection framework integrating concept factorization with robust label learning. Unlike traditional unsupervised concept factorization-based feature selection methods, SCFLR incorporates label information through a regression-based loss function derived from concept vectors and employs an $L_{2,1}$-norm regularization scheme to enhance robustness against noisy or imperfect labels. In addition, dual-graph regularization is adopted to preserve local geometric structures in both feature and sample spaces. Extensive experiments on multiple image datasets demonstrated that SCFLR achieves superior performance compared to several state-of-the-art semi-supervised feature selection methods.

Despite the promising performance of these hybrid, two-stage, and semi-supervised feature selection methods, several challenges remain in high-dimensional settings. Many existing approaches still suffer from considerable redundancy in the selected feature subsets, limited global search capability, and high computational cost when processing large-scale datasets. Moreover, premature convergence and insufficient exploration often prevent the algorithms from consistently identifying optimal or near-optimal feature subsets. To address these limitations, the proposed TMPA-HC algorithm introduces a two-stage framework that combines initial Fisher-based filtering with a multi-subpopulation co-evolution strategy, enhanced by adaptive control, subpopulation cooperation, and local refinement mechanisms. This design aims to improve search efficiency, maintain population diversity, and achieve robust feature selection performance across high-dimensional biomedical datasets.

## The proposed approach

This section presents TMPA-HC, a two-stage heterogeneous multi-population optimization framework designed to handle high-dimensional datasets. The core design motivation of TMPA-HC is to address the intrinsic conflict between exploration efficiency and solution refinement under extremely high-dimensional and small-sample settings.

To this end, TMPA-HC adopts a structured cooperative strategy consisting of three tightly coupled components: (i) explicit functional specialization of heterogeneous subpopulations to decouple distinct search behaviors, (ii) controlled information exchange mechanisms to enable cross-population knowledge sharing without homogenization, and (iii) adaptive regulation mechanisms driven by evolutionary feedback to stabilize convergence across different search stages. Together, these components form a unified framework that balances global exploration, local exploitation, and long-term robustness in complex feature selection landscapes.

### Solution representation and fitness evaluation

Each individual is encoded as a continuous vector, formulated in Eq. (1). Each element in the vector is a real-valued variable bounded in [0, 1].

1 $$\begin{aligned} x_i = (x_{i1}, x_{i2}, ..., x_{ij} ..., x_{in}). \end{aligned}$$

where $x_i$ represents the *i*-th solution, $x_{ij}$ refers to the *j*-th feature of $x_i$, and *n* indicates overall number of features.

However, the solutions in continuous space described above cannot be directly applied to the feature selection problem. The continuous representation is mapped to a binary mask $b_i \in \{{0, 1}\}$ using a S-shaped transfer function *ST* as Eq. (2) - Eq. (3). The S-shaped transfer function provides a smooth probabilistic mapping between continuous search dynamics and binary feature selection decisions, allowing gradual changes in continuous variables to translate into adaptive feature inclusion probabilities. This property is particularly beneficial when combined with stochastic perturbation mechanisms, as it avoids overly abrupt binary flips and helps preserve search stability.

2 $$\begin{aligned} ST(x_{ij}) = \dfrac{1}{1 + exp(-x_{ij}/2)}. \end{aligned}$$

3 $$\begin{aligned} b_{ij} = {\left\{ \begin{array}{ll} 1, & ST(x_{ij})> rand,\\ 0, & \text {otherwise}. \end{array}\right. } \end{aligned}$$

where $b_{ij}$ indicates whether to select this feature, rand is the random number, *j* indexes the original features. The selected feature subset is shown in Eq. (4).

4 $$\begin{aligned} S(x_i) = \{ j | b_{ij} = 1 \}. \end{aligned}$$

For example, if $b_i = (0, 1, 1, 0, 1)$, then the selected feature subset is $S(x_i)=\{2,3,5\}$.

TMPA-HC jointly considers classification accuracy and feature subset size by formulating them into a single scalar fitness function, as shown in Eq. (5).

5 $$\begin{aligned} f(x_i)=\alpha \left( 1-\textrm{Acc}(x_i)\right) +(1-\alpha )\dfrac{|S(x_i)|}{n}. \end{aligned}$$

where $\textrm{Acc}(x_i)$ denotes the 5-fold cross-validated classification accuracy evaluated by an SVM classifier, which is used to measure the predictive performance of the selected feature subset, $|S(x_i)|$ is the number of selected features, and $\alpha \in [0,1]$ controls the trade-off between predictive performance and feature subset size. Minimizing $f(x_i)$ encourages the selection of a compact feature subset while maintaining high classification accuracy. In this study, a large value of $\alpha$ is employed to emphasize classification performance, reflecting practical scenarios in which predictive accuracy is the primary objective and feature reduction acts as a regularizing constraint rather than a dominant goal. By assigning a non-zero weight to the subset size term, the formulation still penalizes unnecessarily large feature sets, guiding the search toward compact yet high-performing solutions.

### Fisher-based filter

In the first stage, a Fisher-based filter is employed as a coarse-grained dimensionality reduction step to suppress highly redundant and noisy features prior to wrapper-based optimization. High-dimensional biomedical datasets often contain a large proportion of features with weak or irrelevant discriminative power, which can significantly hinder evolutionary search efficiency and stability. The Fisher filter serves to reduce this burden by eliminating evidently uninformative dimensions while preserving class-discriminative structures in the data.

The Fisher score of feature $x_i$ is defined as Eq. (6).

6 $$\begin{aligned} \textrm{FS}(x_i) = \frac{\sum _{c=1}^{C} n_c \left( \mu _{c,i} - \mu _i \right) ^2}{\sum _{c=1}^{C} n_c \sigma _{c,i}^2}. \end{aligned}$$

The pseudo-code describing the Fisher filtering strategy is provided in Algorithm 1. With the dataset and the specified feature subset size |*S*|, class-dependent statistics are employed to evaluate the Fisher score of each feature $x_i$. For each feature, both between-class variance and within-class variance are estimated, and their ratio is employed as the corresponding Fisher score. According to the specified subset size |*S*|, features are ranked in descending order of their Fisher scores, and the top features are selected to form the initial subset $S_1$, which is subsequently passed to the second stage of the algorithm.

**Algorithm 1:**
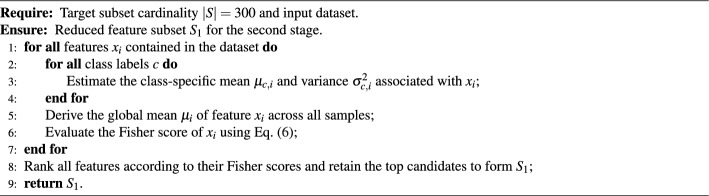
Fisher-based feature filtering.

### Multi-population framework

The entire population *P* of size *NP* is evenly divided into three heterogeneous subpopulations as Eq. (7).

7 $$\begin{aligned} P = P_{exp} \cup P_{expn} \cup P_{bal}, \qquad |P_{exp}| = |P_{expn}| = |P_{bal}| = \frac{NP}{3}. \end{aligned}$$

The division into three explicitly heterogeneous subpopulations is intentionally designed to decouple conflicting search behaviors that are difficult to balance within a single homogeneous population, especially under high-dimensional feature selection scenarios. Rather than relying solely on parameter adaptation to implicitly regulate exploration and exploitation, TMPA-HC assigns distinct functional roles to separate subpopulations, allowing each group to focus on a well-defined search objective while cooperating at the population level. Each subpopulation plays a distinct role: Exploration subpopulation: long-range search and diversity preservation, Exploitation subpopulation: fine-grained local refinement, Balance subpopulation: adaptive mixture of exploration and exploitation. From a structural perspective, the three-subpopulation design can be viewed as a minimal functional decomposition that explicitly decouples conflicting search objectives while maintaining manageable coordination complexity.

The choice of three subpopulations is motivated by the fundamental decomposition of search behaviors in metaheuristic optimization into exploration, exploitation, and transitional regulation. While a two-subpopulation configuration can represent exploration and exploitation, it lacks an explicit mechanism to stabilize their interaction, often leading to oscillatory or unbalanced search dynamics. Introducing a third, balance-oriented subpopulation provides an intermediate regulatory layer that mitigates excessive divergence or premature convergence.

Let $x_{k,\textrm{best}}$ denote the best individual in subpopulation $P_k$, where $k \in \{\textrm{exp}, \textrm{expn}, \textrm{bal}\}$. The global best solution found so far is denoted by $x_{\textrm{best}}$.

TMPA-HC employs a bi-adaptive scaling mechanism where each subpopulation dynamically adjusts its exploration strength according to its historical success rate and the evolutionary progress, enabling stable early-stage diversification and late-stage convergence. A subpopulation-specific scaling factor is defined as Eq. (8).

8 $$\begin{aligned} F_k = F_k^{\textrm{base}} \left( 0.5 + 0.5R_k\right) \left( 0.4 + 0.6p\right) , \qquad p = 1 - \dfrac{t}{T_{\max }}. \end{aligned}$$

where $R_k$ is the success ratio, *p* is evolutionary progress, which linearly decreases with iteration count to modulate the transition from global exploration to local exploitation, $k \in \{ exp, expn, bal\}$. Here, the success ratio $R_k$ reflects the recent effectiveness of a given subpopulation, enabling feedback-driven adjustment of its search intensity, while the progress term *p* provides a global evolutionary schedule that gradually shifts the overall search from diversification to convergence. By combining these two factors multiplicatively, TMPA-HC integrates both short-term performance feedback and long-term evolutionary trends into its adaptive control strategy.

#### Exploration subpopulation

The exploration subpopulation is exclusively responsible for discovering new and potentially unexplored regions of the search space. To maximize search diversity, this subpopulation deliberately weakens direct convergence pressure from global optima and instead emphasizes stochastic directional perturbations and long-range exploratory moves. Its update mechanism is therefore designed to prioritize diversity preservation and large-step exploration rather than immediate fitness improvement.

To maintain high diversity, a dimension-wise random mutation is further applied, allowing certain variables to be reassigned within the full range of bounds. Together, these components equip the Exploration Subpopulation with strong global exploration capability, ensuring effective traversal of large and complex search landscapes. For individual $x_i \in P_{exp}$, TMPA-HC performs as Eq. (9).

9 $$\begin{aligned} v_i = x_i + F_{\textrm{exp}}\, r\odot \big (x_{r1}-x_{r2}\big ) + 0.5\,F_{\textrm{exp}}\cdot \textrm{Levy}(n) + \lambda \big (x_{\textrm{pbest}} - x_i\big ). \end{aligned}$$

where $r \sim \mathscr {U}(0,1)^n$, $x_{r1}, x_{r2} \in P_{exp}$, *Levy*(*n*) is Lévy-flight step vector, $x_{pbest}$ is sampled from the top $p_{best}$ proportion of the population, $\lambda$ controls elite attraction.

To further enhance search diversity, a dimension-wise random mutation is applied to the individuals in exploration subpopulation, mimicking biological point mutations by randomly altering individual components and enabling the exploration subpopulation to escape early convergence as Eq. (10).

10 $$\begin{aligned} v_{ij} = {\left\{ \begin{array}{ll} \mathscr {U}(L_j,U_j), & \text {with probability } p_{\textrm{mut}},\\ v_{ij}, & \text {otherwise}. \end{array}\right. } \end{aligned}$$

This subpopulation provides global and exploratory movement with heavy-tailed jumps.

#### Exploitation subpopulation

The exploitation subpopulation focuses on intensifying the search around high-quality regions identified during evolution. Unlike the exploration group, this subpopulation places strong emphasis on convergence-driving information, such as global and subpopulation elites. Its update strategy is specifically tailored to perform fine-grained local refinement while maintaining controlled stochasticity to avoid shallow local optima, as shown in Eq. (11).

11 $$\begin{aligned} v_i = x_i + F_{\textrm{expn}}\, r \odot \big (x_{\textrm{best}} - x_i\big ) + 0.5\,F_{\textrm{expn}} \big (x_{\textrm{expn},\textrm{best}} - x_i\big ) + \lambda \big (x_{\textrm{pbest}} - x_i\big ). \end{aligned}$$

where $x_{\textrm{best}}$ denotes the global best solution found so far, $x_{\textrm{expn},\textrm{best}}$ denotes the best individual in the exploitation subpopulation, $r \sim \mathscr {U}(0,1)^n$, and $\lambda$ controls the strength of elite attraction.

To avoid becoming trapped in shallow local optima, a Gaussian perturbation is applied to the updated vector, as shown in Eq. (12). This perturbation introduces controlled stochastic variability around promising solutions, improving local search capability while preserving convergence stability.

12 $$\begin{aligned} v_i \leftarrow v_i + \mathscr {N}\big (0, \sigma \,(U-L)\big ). \end{aligned}$$

#### Balance subpopulation

The balance subpopulation acts as an adaptive intermediary between global exploration and local exploitation. Rather than committing to a single search behavior, it dynamically integrates exploratory perturbations and exploitative guidance within each update step as Eqs. (13) - (15). This design allows the balance group to stabilize population dynamics and mitigate excessive polarization between exploration-dominant and exploitation-dominant behaviors.

13 $$\begin{aligned} E_i= & F_{\textrm{bal}}\, r \odot \big (x_{r1}-x_{r2}\big ) + F_{\textrm{bal}}\cdot \textrm{Levy}(n). \end{aligned}$$

14 $$\begin{aligned} D_i = F_{\textrm{bal}}\, r \odot \big (x_{\textrm{best}}-x_i\big ). \end{aligned}$$

15 $$\begin{aligned} v_i= & \dfrac{1}{2}\big (x_i+E_i\big ) + \dfrac{1}{2}\big (x_i+D_i\big ) + \lambda \big (x_{\textrm{pbest}}-x_i\big ). \end{aligned}$$

To prevent premature convergence while preserving stability, moderate Gaussian noise is applied as Eq. (16), functioning as small biological fluctuations that help sustain adaptability. Through this hybrid update mechanism, the Balance Subpopulation continuously adjusts its search behavior, acting as a buffering layer that stabilizes the algorithm’s overall dynamics and maintains diversity across generations.

16 $$\begin{aligned} v_i \leftarrow v_i + \mathscr {N}\big (0, \sigma \,(U-L)\big ). \end{aligned}$$

### Cross-subpopulation cooperation mechanisms

#### Heterosis-driven hybridization

The heterosis-driven hybridization mechanism aims to enhance population diversity and accelerate information exchange across subpopulations. From an algorithmic perspective, this operation can be interpreted as a controlled cross-subpopulation recombination strategy that merges complementary search information preserved in different subpopulations, thereby accelerating information diffusion without forcing population homogenization. Every $T_{hyb}$ iterations, TMPA-HC selects elite individuals from different subpopulations and forms cross-population mating pairs. Each pair generates a hybrid solution through a weighted combination of the parents, as shown in Eq. (17). This operation mimics biological heterosis, where offspring produced by genetically diverse parents often exhibit superior traits due to complementary gene interactions.

17 $$\begin{aligned} h = r\, x_p^{(a)} + (1-r)\, x_p^{(b)}, \qquad r\sim \mathscr {U}(0,1). \end{aligned}$$

After the hybrid is generated, its fitness is compared with that of both parents. If the hybrid provides a better solution, it replaces the inferior parent according to Eq. (18). This selective replacement ensures that beneficial genetic material propagates through the population, while ineffective combinations are discarded. Through this process, the algorithm strengthens promising regions, improves global exploration, and maintains robust evolutionary dynamics across all subpopulations.

18 $$\begin{aligned} \text {Replace if }\; f(h) < \min \{f(x_p^{(a)}),\, f(x_p^{(b)})\}. \end{aligned}$$

where $x_p^{(a)}$ and $x_p^{(b)}$ denote elite parents sampled from two different subpopulations. This mechanism accelerates information flow and exploits heterosis effects.

#### Subpopulation reorganization

The worst-performing subpopulation is periodically reorganized to restore diversity and prevent long-term stagnation. Let $P_w$ denote the subpopulation with the highest mean fitness. The best individual in this subpopulation, denoted by $x_{w,\textrm{best}}$, is selected as a seed to regenerate new candidate solutions:

19 $$\begin{aligned} x_i \leftarrow x_{w,\textrm{best}} + 0.25\,(u-0.5)\odot (U-L), \qquad u\sim \mathscr {U}(0,1)^n. \end{aligned}$$

This reorganization strategy introduces controlled diversity while preserving promising structural information inherited from the subpopulation elite.

#### Cooperation-based information transfer

A cyclic donor–receiver strategy is executed every $T_{\text {coop}}$ iterations.Let $x_{\textrm{worst}}^{(j)}$ denote the worst-performing individual in the receiver subpopulation $P_j$.

20 $$\begin{aligned} x_{\textrm{worst}}^{(j)} \leftarrow 0.7\, x_{\textrm{best}}^{(i)} + 0.3\, x_{\textrm{worst}}^{(j)} + \mathscr {N}\big (0,\sigma (U-L)\big ). \end{aligned}$$

This promotes coordinated progress among subpopulations.

### Local search, restart, and adaptive control

#### Elite local intensification

Top-ranked individuals undergo elite-based Gaussian search:

21 $$\begin{aligned} x_ix2019; = x_i + \mathscr {N}\big (0, \rho \,(U-L)\big ). \end{aligned}$$

where $\rho = \rho _0 \left( 1 - \frac{t}{T_{\max }}\right)$. Local intensification enhances fine precision near high-potential solutions.

#### Stagnation-aware restart

To prevent the evolutionary process from becoming trapped in long-term stagnation, TMPA-HC incorporates a stagnation-aware restart mechanism. When the global best fitness fails to improve for $T_{stag}$ consecutive iterations, a portion of the worst-performing individuals is regenerated in the vicinity of the global best solution. This is achieved by adding a scaled Gaussian perturbation to the global best, as shown in Eq. (22).

22 $$\begin{aligned} x_i \leftarrow x_{\textrm{best}} + \delta \cdot \mathscr {N}\big (0,\sigma (U-L)\big ). \end{aligned}$$

The scaling coefficient $\delta$ shrinks gradually as evolution progresses,

23 $$\begin{aligned} \delta = \max \left( 0.05,\,1-\dfrac{t}{T_{\max }}\right) . \end{aligned}$$

From an optimization standpoint, this restart mechanism functions as a diversity recovery operator that selectively rejuvenates degraded individuals while preserving convergence momentum, rather than performing disruptive full-population reinitialization. This design ensures wide-range restarts in early iterations and more localized refinements near the end. Biologically, this mechanism resembles population recovery after environmental bottlenecks, where a surviving elite group seeds new generations. By periodically refreshing degraded individuals, the algorithm mitigates premature convergence and maintains search vitality in high-dimensional spaces.

#### Success-rate–driven adaptive control

TMPA-HC dynamically adjusts the evolutionary intensity of each subpopulation through a success-rate monitoring mechanism. For subpopulation *k*, the algorithm records how many trial individuals successfully replace their parents and computes the success ratio:

24 $$\begin{aligned} R_k = \dfrac{\text {success}_k}{\text {success}_k + \text {failure}_k + \epsilon }. \end{aligned}$$

This statistic provides a real-time estimate of how effectively a subpopulation is exploring its search region. The value of $R_k$ is incorporated into an exponential moving memory, which subsequently modulates each subpopulation’s scaling factor via Eq. (8). As a result, subpopulations with high success rates receive stronger search intensities, while ineffective ones are dampened to avoid wasting exploration budget. This adaptive strategy enables TMPA-HC to self-regulate its exploration–exploitation balance throughout evolution, analogous to adaptive behavioral modulation in biological species reacting to environmental feedback.

**Fig. 1:**
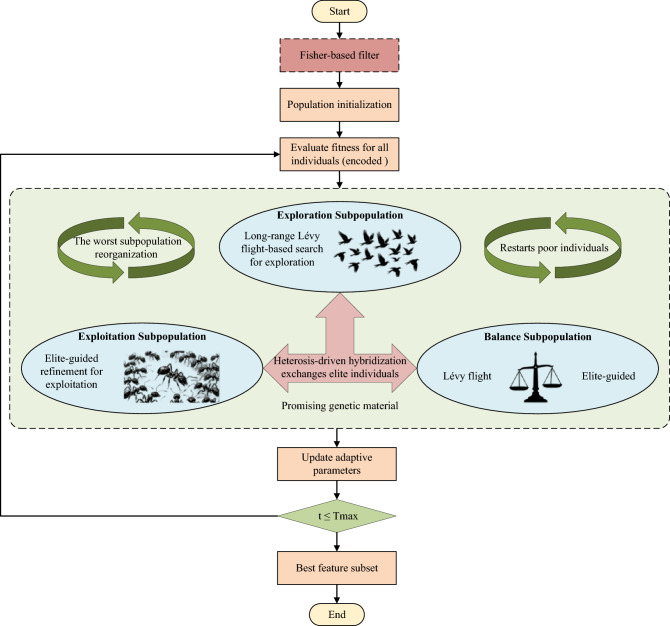
The flowchart of TMPA-HC algorithms for feature selection.

### Greedy selection

After generating trial solutions, TMPA-HC applies a greedy replacement strategy to update the population. For each individual, the trial vector replaces the current solution only if it yields a strictly lower fitness:

25 $$\begin{aligned} x_i \leftarrow {\left\{ \begin{array}{ll} v_i, & \text {if } f(v_i) < f(x_i),\\ x_i, & \text {otherwise}. \end{array}\right. } \end{aligned}$$

This deterministic approach ensures that population quality monotonically improves or remains stable across iterations. It also guarantees that beneficial evolutionary changes propagate immediately, while inferior updates are effectively filtered out. The greedy mechanism is simple yet efficient and is widely used in differential-evolution–based metaheuristics because of its strong convergence-driving effect.

### Computational complexity

The computational complexity of TMPA-HC is mainly dominated by the wrapper-based fitness evaluation, which involves training and testing a classifier. Given a population size *NP* and a maximum iteration number $T_{\max }$, the overall computational complexity is given by:

26 $$\begin{aligned} \mathscr {O}\big (T_{\max } \cdot NP \cdot C_{\text {fit}}\big ), \end{aligned}$$

where $C_{\text {fit}}$ denotes the computational cost of evaluating a candidate feature subset using the selected classifier. In the case of SVM-based fitness evaluation, $C_{\text {fit}}$ depends on both the dimensionality of the chosen subset and the number of training samples, and can grow significantly for high-dimensional datasets. Since fitness evaluations are independent across individuals, TMPA-HC naturally supports parallel implementation, allowing substantial reductions in wall-clock runtime without altering the algorithmic complexity.

### Pseudocode

The overall procedure of TMPA-HC is summarized in Algorithm 2, and the algorithm flowchart is shown in Fig. 1. The algorithm begins by initializing a continuous population and dividing it into three heterogeneous subpopulations–exploration, exploitation, and balance–each responsible for a different search behavior. Once all individuals have been evaluated, TMPA-HC proceeds with an iterative evolutionary process that continues until the maximum iteration count is achieved.

At each iteration, the three subpopulations are updated independently using their respective operators: long-range Lévy-based search for exploration, elite-guided refinement for exploitation, and a mixed strategy for the balance group. A greedy selection mechanism then evaluates trial individuals and updates the global best solution. Several cooperative mechanisms are periodically triggered to enhance cross-population information flow and maintain search robustness. Heterosis-driven hybridization exchanges elite structures between different subpopulations. Subpopulation reorganization regenerates the worst-performing group to restore diversity. Local intensification applies Gaussian refinement to high-quality individuals, and cooperation-based transfer propagates promising genetic material across subpopulations. When stagnation is detected, TMPA-HC partially restarts poor individuals near the global best to escape premature convergence. Throughout evolution, success-rate statistics are continuously collected to adaptively update the control parameters of each subpopulation. After all iterations are completed, the best-performing individual is mapped to a binary mask and returned as the final feature subset.

**Algorithm 2:**
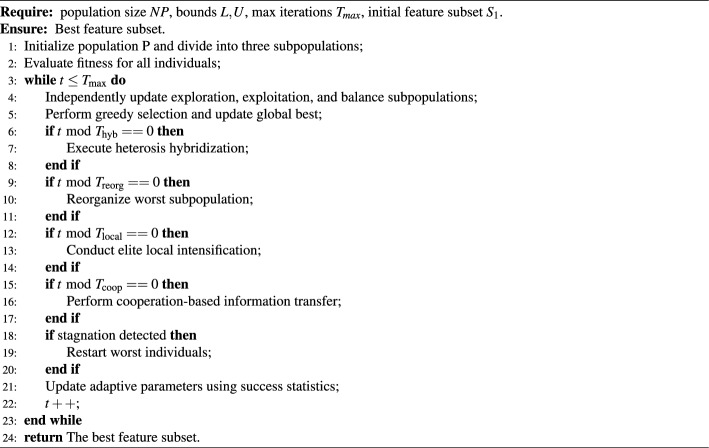
TMPA-HC for feature selection.

## Experimental design

A set of experiments were conducted to rigorously evaluate the proposed algorithm’s performance, which can be grouped into the following six categories:

1. Assessment of TMPA-HC on continuous optimization problems: The performance of TMPA-HC in continuous search spaces was evaluated using the CEC2022 benchmark suite as standard test functions.
2. Performance evaluation of TMPA-HC on high-dimensional biomedical data: The classification effectiveness of TMPA-HC was extensively investigated on 12 publicly available high-dimensional gene expression datasets obtained from biomedical microarray repositories.
3. Classifier comparison analysis: To evaluate the robustness of TMPA-HC with respect to the choice of classifier, comparative experiments were conducted by integrating different classifiers into the wrapper evaluation stage, enabling an assessment of classification stability and generalization consistency under various learning models.
4. Parameter sensitivity and robustness analysis: To investigate the influence of key control parameters on algorithmic performance and validate the rationality of the default settings, a series of controlled experiments were carried out by systematically varying critical representative parameters. This allows for a quantitative evaluation of the algorithm’s robustness, stability, and reproducibility with respect to its core cooperative mechanisms.
5. Ablation-based component analysis of TMPA-HC: To examine the role and impact of each algorithmic module, a series of ablation experiments were performed on multiple high-dimensional biomedical datasets by selectively deactivating specific strategies embedded in TMPA-HC.
6. Evolutionary dynamics and operator attribution analysis: To enhance the explainability of the proposed method and provide deeper insights into its search behavior, an operator attribution analysis (EvoMapX) was conducted^62^. This experiment visualizes the dynamic contribution of each heterogeneous subpopulation, verifying the rationality of the cooperative framework.

The computations were performed on a Windows 10 workstation equipped with an Intel Core i9-13900K processor and 32 GB of memory. All methods were written in Python.

### Benchmark algorithms and experimental setup

To evaluate the performance of TMPA-HC, experiments were first conducted using the CEC2022 test functions, which include one unimodal function, four basic functions, three hybrid functions, and four composite functions. For benchmarking purposes, a set of widely used optimization algorithms was selected as baselines, including GA^63^, FPA^64^, PSO^65^, GWO^66^, HHO^67^, SSA^68^, WOA^69^, SMA^70^, JAYA^71^, the Rime Optimization Algorithm (RIME)^72^, RUN^73^, and the Honey Badger Algorithm (HBA)^74^, Tom and Jerry optimization (TJO)^75^, Poor and Rich optimization (PRO)^76^, Rao algorithms^77^. The specific parameter settings for each comparison algorithm are provided in Table 1. To ensure fair and statistically reliable comparisons, all algorithms were executed with a uniform population size of 30, a maximum of 1000 iterations, and 30 independent runs for each 20-dimensional function, and the random seed was initialized to 42.

In addition, the performance of TMPA-HC on high-dimensional feature selection tasks was further examined using 12 biomedical datasets listed in Table 2. TMPA-HC was compared against several competitive feature selection approaches, including SC-MBO-BLS^52^, TSHFSACO^53^, IGWO^54^, TSMMOPSO-F1^55^, as well as a group of algorithms enhanced with the same Fisher filter, namely F-GA, F-GWO, F-HBA, F-HHO, F-JAYA, F-RIME, F-SSA, F-WOA, F-TJO, F-PRO, and F-Rao. The internal parameter settings of these methods followed either those reported in the original studies or the configurations listed in Table 1. Each algorithm was run independently 30 times on every dataset, using a population of 30 and at least 100 iterations, and the random seed was initialized to 42. For all filter-enhanced algorithms, the Fisher filter was applied using the same configuration as TMPA-HC, with the feature subset size fixed at $|S|=300$.

To ensure the reliability and reproducibility of the experimental results, all high-dimensional biomedical datasets were evaluated using a 5-fold cross-validation (5-CV) protocol, which is a standard and rigorous evaluation method for small-sample high-dimensional data scenarios (consistent with the characteristics of the gene expression datasets in this study). In each fold of cross-validation, the dataset was randomly partitioned into a training set (80% of the samples) and an independent test set (20% of the samples) while maintaining the original class distribution (stratified sampling). Importantly, to prevent any data leakage, all stages of the proposed pipeline–including Fisher-score-based feature filtering, wrapper-based feature selection optimization, and SVM classifier training–were performed exclusively on the training fold. The test fold remained completely unseen until the final evaluation of classification accuracy. This cross-validation strategy effectively alleviates the overfitting risk caused by the small sample size of the biomedical datasets and ensures the fairness of performance comparison among all competing algorithms.

In the classifier comparison analysis, besides the default SVM classifier adopted in TMPA-HC, XGBoost (XGB) was incorporated into the wrapper evaluation framework to examine the robustness of the selected feature subsets under different classification paradigms. For XGBoost, the number of trees was set to 100, the maximum tree depth to 6, the learning rate to 0.1, and the subsampling ratio to 0.8. For fairness, the same cross-validation protocol and evaluation metric were applied to all classifiers throughout the comparative experiments. All parameter settings followed commonly adopted configurations in the literature to avoid introducing additional tuning bias.

For the parameter sensitivity analysis, to balance comprehensive evaluation with computational feasibility, we selected two representative datasets with different characteristics: *D*2 (medium dimension) and *D*9 (high dimension). We focused on the parameters most critical to the proposed cooperative and adaptive mechanisms. Specifically, representative control parameters of TMPA-HC, including the hybridization interval $T_{hyb}$, cooperation interval $T_{coop}$, restart threshold $\rho _{\textrm{restart}}$, and elite influence factor $\lambda$, were individually varied within predefined ranges: $T_{hyb} \in \{2, 5, 10, 20\}$, $T_{coop} \in \{3, 6, 12, 24\}$, $\rho _{\textrm{restart}} \in \{0.1, 0.2, 0.3, 0.4\}$, and $\lambda \in \{0.3, 0.5, 0.7, 0.9\}$. All other parameters were kept fixed at their default values shown in Table 1 during these tests (One-Factor-At-A-Time strategy). Each configuration was independently executed 30 times under the same evaluation protocol. The sensitivity results were analyzed in terms of classification accuracy to investigate the robustness of TMPA-HC with respect to parameter perturbations.

The datasets in Table 2 were obtained from publicly available high-dimensional biomedical microarray repositories based on gene expression profiles^78,79^, UCI Machine Learning Repository. Such datasets are typically generated using high-throughput techniques, including microarray analysis and next-generation sequencing, which enable the simultaneous measurement of expression levels for thousands of genes. These data resources are extensively employed in biomedical applications such as disease classification, biomarker identification, and drug discovery. By examining variations in gene expression patterns, valuable insights into disease-related molecular mechanisms can be obtained, thereby supporting the development of effective therapeutic strategies.

**Table 1:** Parameter settings.

Algorithm	Parameter Setting
TMPA-HC	$T_{hyb} = 5$, $T_{reorg} = 12$, $T_{local} = 8$, $r_l = 0.12$, $p_{\textrm{best}} = 0.2$, $T_{coop} = 6$, $T_{\textrm{stag}} = 30$, $\rho _{\textrm{restart}} = 0.2$, $\alpha _e = 2.0$, $p_e = 0.3$, $\alpha _x = 0.5$, $p_x = 0.1$, $\alpha _b = 1.0$, $p_b = 0.2$, $\sigma = 0.05$, $\beta = 1.5$, $\lambda = 0.7$, $C = 4$.
GA	$CR = 1$, $MR = 0.01$.
FPA	$p = 0.8$, $\beta = 1.5$, $u \sim \mathscr {N}(0, \sigma _u^2)$, $v \sim \mathscr {N}(0, 1)$.
PSO	$w \in [w_{\min }, w_{\max }]$ with $w_{\max }=0.9$, $w_{\min }=0.2$, $c_1 = c_2 = 2$, $v_{\max } = 6$, $r_1, r_2 \sim \mathscr {U}(0,1)^n$.
GWO	$\alpha \in [0,2]$.
HHO	$E_1 = 2(1 - t/T)$, $E_0 \sim \mathscr {U}(-1,1)$, $q, r \sim \mathscr {U}(0,1)$, $J = 2(1-r_3)$, $\beta = 1.5$, $r_1, r_2, r_3 \sim \mathscr {U}(0,1)$.
SSA	$c_1 = 2e^{-(\frac{4t}{T})^2}$, $c_2 \sim \mathscr {U}(0,1)^n$, $c_3 \sim \mathscr {U}(0,1)^n$.
WOA	$a \in [0,2]$, $A = 2 a r_1 - a$, $C = 2 r_2$, $b=1$, $l \in [1,a_2]$, $p \in [0,1]$.
SMA	$z = 0.03$, $a = \operatorname {arctanh}\!\left( 1 - \frac{t}{T}\right)$, $b = 1 - \frac{t}{T}$, $p = \tanh \!\left( |f_i - f_{\textrm{best}} |\right)$, $v_b \sim \mathscr {U}(-a,a)^n$, $v_c \sim \mathscr {U}(-b,b)^n$.
JAYA	–
RIME	$W = 5$.
RUN	$f = 20 \exp (-12 \, t/T)$, $SF = 2 (0.5 - r) \cdot f$, $r \sim \mathscr {U}(0,1)$, $\gamma = r_\gamma \cdot (\textbf{x}_i - r_\gamma ' (\textbf{u} - \textbf{x}_i)) \exp (-4\, t/T)$, $g \sim \mathscr {U}(0,2)$, $\mu = 0.5 + 0.1 \, \mathscr {N}(0,1)$, $r, u \sim \mathscr {U}(0,1)^n$, $w \sim \operatorname {unifrnd}(0,2)$, $C \sim \{0,1\} \cdot (1 - r)$.
HBA	$C = 2$; $\beta = 6$, $F \in \{-1,1\}$.
TJO	–
PRO	–
Rao	–

**Table 2:** Datasets considered in the feature selection experiments of TMPA-HC.

ID	Dataset Name	Num of Instances	Num of Features	Num of Classes
*D*1	colon	62	2000	2
*D*2	GLIOMA	50	4434	4
*D*3	leukemia 1	72	5327	3
*D*4	DLBCL	77	5469	2
*D*5	TOX_171	171	5748	4
*D*6	ALLAML	72	7129	2
*D*7	leukemia 2	72	7129	4
*D*8	Prostate Tumors	102	10509	2
*D*9	CLL_SUB_111	111	11340	3
*D*10	Prostate_GE	34	12600	2
*D*11	SMK_CAN_187	187	19993	2
*D*12	GLI_85	85	22283	2

To comprehensively assess both the independent effectiveness of each core mechanism and the rationality of critical structural design choices in TMPA-HC, a unified ablation and structural validation strategy was employed. Specifically, one algorithmic component was disabled at a time while preserving all remaining settings identical to those of the complete TMPA-HC, thereby allowing a direct quantification of each module’s contribution to the overall optimization performance. In addition to mechanism-level removal experiments, structural comparison tests were conducted by varying the number of subpopulations to verify the necessity of the tri-subpopulation configuration adopted in TMPA-HC. All experiments were carried out on several high-dimensional biomedical datasets summarized in Table 2. As detailed in Table 3, each variant corresponds to a specific removal or structural modification strategy. For fairness and comparability, all ablation and structural variants employed the same parameter settings, population size, termination conditions, and evaluation protocols as the original TMPA-HC.

**Table 3:** Ablation variants of TMPA-HC and corresponding removal strategies.

Algorithm	Removed Component	Description
TMPA-HC	None.	Complete proposed algorithm.
TMPA-HC–LF	Lévy-flight mechanism.	All Lévy-flight perturbation terms are removed from exploration and balance subpopulations.
TMPA-HC–AD	Adaptive control mechanism.	The success-rate–driven adaptive scaling is disabled and fixed control parameters are used.
TMPA-HC–CO	Cross-subpopulation cooperation.	Heterosis-driven hybridization and cooperation-based information transfer are removed.
TMPA-HC–REO	Subpopulation reorganization.	The periodic reorganization of the worst-performing subpopulation is disabled.
TMPA-HC–RS	Restart and local search.	Stagnation-aware restart and elite local intensification mechanisms are removed.
TMPA-HC–2SP	Two-subpopulation configuration.	The population is divided into only exploration and exploitation groups without the balance subgroup.
TMPA-HC–4SP	Four-subpopulation configuration.	An additional subpopulation is introduced to further diversify search behaviors.
MPA-HC	Fisher-based filter.	The Fisher-score–based feature filtering step is removed, and the original MPA-HC framework operates directly on the full feature space without preliminary feature screening.

For the evolutionary dynamics and operator attribution analysis, we selected two representative datasets to visualize the algorithm’s internal behavior: *D*2 (GLIOMA, representing medium-dimensional data) and *D*9 (CLL_SUB_111, representing high-dimensional data). To capture the search trajectory, we recorded the real-time success rate ($R_k$) of each subpopulation (Exploration, Exploitation, and Balance) at every iteration during the optimization process. This analysis followed the same parameter settings and evaluation protocols as the main feature selection experiments, ensuring that the visualized dynamics faithfully reflect the algorithm’s actual performance.

## Experiments and discussion

### Results obtained on the CEC2022 benchmark functions

**Fig. 2:**
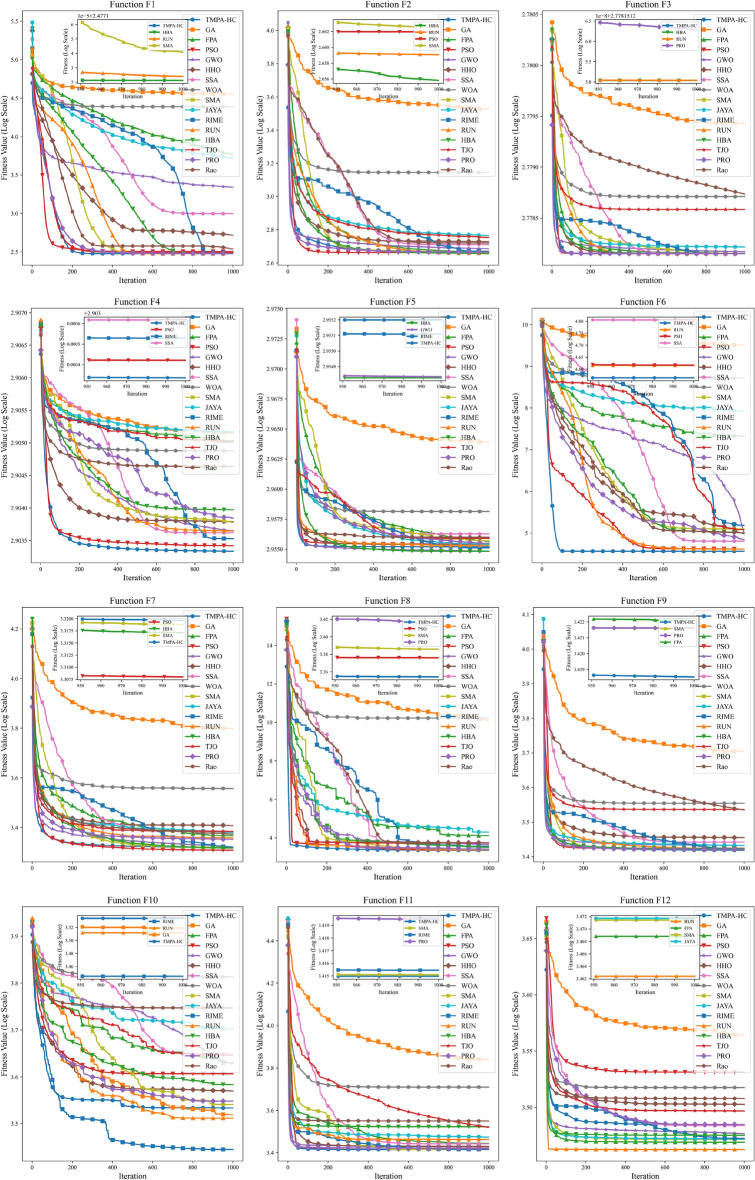
Convergence behavior of different algorithms over benchmark functions of the CEC2022.

As illustrated in Fig. 2, TMPA-HC achieves superior results on the majority of benchmark functions. In particular, for a subset of test functions including *F*1, *F*3, *F*4, *F*6, *F*8, *F*9, and *F*11, it produces solutions closer to the global optima compared with other algorithms. TMPA-HC also demonstrates competitive outcomes on function *F*5. Concerning convergence behavior, TMPA-HC generally converges faster than the comparison algorithms, showing the most rapid convergence on *F*4, *F*5, *F*6, *F*7, *F*8, and *F*11, while ranking second in speed for *F*1, *F*2, *F*3, and *F*10. Furthermore, across the 12 test functions spanning four categories, TMPA-HC exhibits consistent and stable performance, indicating its effectiveness not only on unimodal and basic functions but also on mixed and composite functions.

The TMPA-HC algorithm exhibits fast and stable convergence performance on a unimodal function through heterogeneous cooperation of three subgroups, low-frequency high-quality mixing, and an adaptive local reinforcement mechanism. The unimodal function in CEC2022 is primarily used to evaluate the algorithm’s convergence performance, including both the precision, speed, and stability of convergence, placing strict demands on its design. In TMPA-HC, the development of the subgroup employs a dual-guided update strategy using both the global optimum and the subgroup optimum, ensuring the search direction always points to the currently known optimal region. Simultaneously, the random vector is used only for step size scaling without changing the search direction, effectively avoiding unnecessary directional perturbations. Furthermore, the adaptive step size decay mechanism gradually reduces the search step size with iterations, enhancing the stability of later search stages. Combined with a local search strategy that gradually shrinks with the iteration process, TMPA-HC can quickly approximate the global optimum and achieve high final accuracy on the unimodal function. This enables the subgroup to achieve stable directional convergence in *F*1. Ultimately, it surpassed the PSO algorithm, which has the second fastest convergence speed, reaching the optimal solution after 300 iterations, while the HBA and RUN algorithms both reached the same optimal solution after 500 iterations.

On the basic functions, TMPA-HC exhibits good global search capabilities and fast convergence speed, mainly due to the effective balance between its exploration and exploitation mechanisms. The basic functions of CEC2022 typically contain a few local optima, requiring the algorithm to possess a certain ability to escape local optima while maintaining high convergence efficiency. TMPA-HC’s exploration subgroup employs an update method combining difference vectors and Lévy flight, introducing moderate long-distance jumps while maintaining directional search capabilities, thereby enhancing its ability to break through local optima. Simultaneously, the introduced p-best guidance mechanism makes random search more targeted, avoiding ineffective exploration. The balancing subgroup, through the linear fusion of exploration and exploitation behaviors, can adaptively and smoothly transition from the exploration phase to the exploitation phase during the search process, thus accelerating overall convergence. These strategies jointly allow TMPA-HC to achieve a balance between global exploration and local exploitation on the basic functions. TMPA-HC achieved optimal values of 600.00 and 800.45 on *F*3 and *F*4 respectively, surpassing other algorithms. Its convergence speed on *F*4 was also very fast, exceeding PSO and maintaining its lead after 100 iterations. However, we can also see that TMPA-HC ranked fourth on *F*5, but its optimized value was only 0.77 different from the first-place HBA algorithm. On *F*2, although it did not converge to the optimal value, its excellent optimization ability resulted in a relatively fast convergence speed, ranking second.

For mixed functions, TMPA-HC maintains stable and competitive search performance, primarily due to its multi-subgroup heterogeneous collaborative search framework. CEC2022’s mixed functions typically consist of multiple subfunctions of different types, with different variable subspaces exhibiting significantly different search characteristics, placing high demands on the algorithm’s adaptability. TMPA-HC addresses this by setting up three complementary subgroups–exploration, development, and balancing–allowing the algorithm to execute different search strategies in parallel during the same iteration, thus adapting to the search needs of different subspaces. Furthermore, the cross-subgroup information transfer mechanism enables timely sharing of superior search information from each subgroup, preventing any subgroup from remaining in low-quality search regions for extended periods. The low-frequency hybridization strategy further promotes the fusion of superior solution structures under different search modes, contributing to improved overall algorithm performance in complex mixed search spaces. TMPA-HC achieved optimal performance on all three mixture functions, reaching $3.66\textrm{E}{+4}$ and $2.26\textrm{E}{+3}$ on *F*6 and *F*8 respectively, surpassing algorithms such as PSO, SMA, and RUN. Furthermore, it achieved the fastest convergence speed on *F*6, reaching the optimal value in just over 100 iterations, far exceeding the convergence speed of PSO and RUN, even though PSO and RUN eventually converged to near their optimal values. However, we can also observe that on *F*7, although TMPA-HC was able to compete with PSO in the early stages (first 400 iterations), it was gradually surpassed by PSO after 400 iterations, indicating that TMPA-HC may have fallen into a local optimum. Nevertheless, TMPA-HC still achieved a fourth-place ranking.

TMPA-HC exhibits strong robustness and stability on combinatorial functions, primarily due to its multi-level diversity maintenance and stagnation handling mechanisms. CEC2022 combinatorial functions typically possess complex multimodal structures and strong deceptiveness, easily leading to premature convergence. TMPA-HC maintains population diversity at multiple levels by exploring Lévy flight within subgroups, subgroup recombination strategies, and stagnation-triggered local restart mechanisms. When the algorithm stagnates during the search process, the restart strategy performs controlled perturbations around the current optimal solution, introducing new search directions while preserving existing search information, thus effectively mitigating premature convergence. Compared to completely random restarts, this strategy enhances the algorithm’s continuous search capability in complex combinatorial functions while ensuring search stability, enabling TMPA-HC to maintain relatively consistent performance on these functions. TMPA-HC achieves optimal convergence values on *F*9 and *F*11, surpassing the second-place SMA by $1.85\textrm{E}{+1}$ and $6.04\textrm{E}{+1}$ respectively. Furthermore, the parameter-less optimization algorithm PRO also achieved a commendable third place on *F*9, with an optimization value of $2.64\textrm{E}{+3}$. Its convergence speed is faster on *F*11, but slower than PSO on *F*9. In *F*10 and *F*12, TMPA-HC ranked fourth and eighth, respectively. It can be seen that in the first 500 iterations of *F*10, only the RIME algorithm escaped the local optimum around the 400th generation, and TMPA-HC also showed a tendency to escape local optima later on. However, the GA and RUN algorithms later outperformed the TMPA-HC method, obtaining better solutions. Its performance in *F*12 was not satisfactory, failing to obtain a good solution for this combinatorial function.

Overall, the CEC2022 benchmark results demonstrate that TMPA-HC exhibits consistent competitiveness across most test functions. Thanks to its heterogeneous multi-subgroup cooperative framework, adaptive exploration-exploitation balance, and multi-level diversity maintenance mechanism, TMPA-HC performs particularly well on unimodal and fundamental functions, exhibiting fast and stable convergence. Furthermore, TMPA-HC maintains robust and stable search performance on more complex mixed and composite functions, indicating its strong adaptability to different optimization scenarios. These results confirm that TMPA-HC is effective not only for relatively simple optimization problems but also demonstrates reliable performance on challenging multimodal and composite problems.

### TMPA-HC high-dimensional feature selection results

**Table 4:** Feature selection result. Values represent the Mean and Standard Deviation (Std) of classification accuracy obtained from 30 independent runs. The best result for each dataset is highlighted in bold.

Dataset	Measure	Methods															
		F-GA	F-GWO	F-HBA	F-HHO	F-JAYA	F-RIME	F-SSA	F-WOA	F-TJO	F-PRO	F-Rao	SC-MBO-BLS	TSHFSACO	IGWO	TSMMOPSO-F1	TMPA-HC
*D*1	Acc	90.12	89.04	89.74	93.67	89.14	89.88	88.46	90.61	89.58	94.12	87.08	92.51	94.48	93.88	90.80	**96.45**
	Std	0.89	0.76	0.96	1.76	0.77	0.86	**0.00**	1.74	1.71	1.33	0.82	1.19	1.05	0.77	1.10	1.26
	Num	149.30	147.67	134.13	16.33	221.83	140.03	223.13	43.40	88.60	**5.33**	48.53	7.40	7.27	5.73	92.33	15.43
	Time	8.21	5.77	40.87	19.99	5.72	22.91	21.50	5.30	10.64	5.52	25.40	38.61	6.05	8.15	4.23	**4.15**
	Rank	9	14	11	5	13	10	15	8	12	3	16	6	2	4	7	**1**
*D*2	Acc	94.67	92.20	92.47	93.07	90.67	93.20	85.93	88.13	89.53	94.07	89.73	88.87	94.07	96.07	93.80	**96.87**
	Std	1.07	1.89	2.11	1.91	1.49	1.83	1.67	2.42	1.84	0.96	2.91	1.98	1.09	1.97	**0.79**	1.23
	Num	174.57	186.67	156.70	58.17	257.83	163.97	234.23	121.50	31.13	19.73	57.57	34.00	19.33	**13.70**	88.60	47.43
	Time	9.85	5.47	32.87	15.66	3.78	17.40	17.31	3.91	10.75	3.42	86.75	35.25	4.74	6.66	3.71	**3.61**
	Rank	3	10	9	8	11	7	16	15	13	5	12	14	4	2	6	**1**
*D*3	Acc	**100.00**	99.81	**100.00**	99.95	99.86	**100.00**	99.49	99.29	86.09	99.60	94.96	96.47	**100.00**	99.16	99.73	**100.00**
	Std	**0.00**	0.49	**0.00**	0.26	0.43	**0.00**	0.67	0.71	2.83	0.61	0.73	0.98	**0.00**	0.85	0.53	**0.00**
	Num	136.93	153.00	134.53	30.03	229.10	134.83	226.07	97.77	64.13	71.60	19.20	18.73	9.97	**8.77**	84.53	43.03
	Time	7.56	5.67	47.22	22.36	5.32	28.35	30.61	5.66	21.29	4.15	4.20	52.94	5.13	9.64	4.91	**4.89**
	Rank	**1**	8	**1**	6	7	**1**	12	12	16	10	15	14	**1**	13	9	**1**
*D*4	Acc	**100.00**	**100.00**	**100.00**	**100.00**	**100.00**	**100.00**	99.91	99.96	99.74	99.96	99.38	99.79	**100.00**	99.64	99.24	**100.00**
	Std	**0.00**	**0.00**	**0.00**	**0.00**	**0.00**	**0.00**	0.33	0.24	0.52	0.22	0.67	0.47	**0.00**	0.59	0.66	**0.00**
	Num	133.30	150.43	131.07	13.87	217.13	134.30	223.00	45.07	5.56	21.93	66.43	22.60	**4.73**	6.00	76.57	24.60
	Time	7.77	5.60	40.44	19.47	5.23	23.88	26.22	4.70	7.36	14.13	**4.11**	47.95	5.04	9.83	5.02	4.84
	Rank	**1**	**1**	**1**	**1**	**1**	**1**	11	10	13	9	15	12	**1**	14	16	**1**
*D*5	Acc	**93.51**	91.59	91.50	89.35	88.48	90.89	85.76	86.68	76.99	82.51	76.74	82.68	77.50	76.08	75.95	91.99
	Std	1.02	1.51	1.35	1.57	**0.92**	1.47	1.29	1.52	1.17	0.81	2.26	1.10	2.05	2.90	1.96	0.97
	Num	225.50	228.97	218.37	137.13	272.10	220.27	248.00	197.73	**11.56**	39.53	22.53	41.73	12.07	21.73	126.93	72.20
	Time	27.84	11.01	120.76	66.52	11.93	89.24	64.38	7.44	5.28	142.13	12.09	100.09	**5.51**	10.57	6.43	11.63
	Rank	**1**	3	4	6	7	5	9	8	13	11	14	10	12	15	16	2
*D*6	Acc	**100.00**	**100.00**	**100.00**	**100.00**	**100.00**	**100.00**	99.95	**100.00**	99.82	**100.00**	99.19	99.15	**100.00**	99.60	**100.00**	**100.00**
	Std	**0.00**	**0.00**	**0.00**	**0.00**	**0.00**	**0.00**	0.26	**0.00**	0.45	**0.00**	0.75	0.73	**0.00**	0.70	**0.00**	**0.00**
	Num	135.47	157.83	127.37	24.37	216.70	133.03	225.60	97.37	8.03	11.03	24.47	27.50	8.53	**8.03**	80.40	40.53
	Time	5.31	3.70	27.92	14.25	3.61	16.34	17.10	3.49	7.36	3.54	5.10	33.05	3.53	6.44	3.80	**3.45**
	Rank	**1**	**1**	**1**	**1**	**1**	**1**	12	**1**	13	**1**	15	16	**1**	14	**1**	**1**

**Table 5:** Continued.

Dataset	Measure	Methods															
		F-GA	F-GWO	F-HBA	F-HHO	F-JAYA	F-RIME	F-SSA	F-WOA	F-TJO	F-PRO	F-Rao	SC-MBO-BLS	TSHFSACO	IGWO	TSMMOPSO-F1	TMPA-HC
*D*7	Acc	93.36	92.29	92.56	93.44	92.81	92.86	92.05	92.70	94.43	93.59	91.90	91.05	**98.55**	94.59	93.36	94.48
	Std	0.61	0.70	0.93	0.84	0.60	0.57	0.60	0.82	0.24	0.63	1.22	1.44	0.69	1.41	0.62	**0.00**
	Num	146.60	150.27	136.70	26.03	223.13	137.47	217.50	83.27	45.50	77.16	19.53	19.37	21.10	**10.90**	85.73	42.87
	Time	6.95	4.97	39.11	19.88	6.88	26.65	29.14	6.25	3.25	4.40	46.33	56.50	5.86	10.39	5.97	**5.78**
	Rank	7	13	12	6	10	9	14	11	4	5	15	16	**1**	2	7	3
*D*8	Acc	96.65	96.11	96.37	97.08	96.24	96.40	96.05	96.30	94.63	97.25	92.79	97.18	97.30	97.80	97.41	**98.20**
	Std	0.52	0.35	0.45	0.71	0.38	0.59	**0.00**	0.50	1.59	0.82	1.92	0.37	0.71	0.65	0.78	0.40
	Num	144.67	156.50	135.10	21.83	223.20	138.77	222.50	58.17	26.80	77.53	73.03	25.17	6.13	**5.70**	87.93	32.63
	Time	7.62	5.68	40.83	21.96	5.61	22.76	22.61	**3.83**	49.65	5.60	31.69	42.27	3.89	7.75	4.26	5.00
	Rank	8	13	10	7	12	9	14	11	15	5	16	6	4	2	3	**1**
*D*9	Acc	**94.67**	92.53	92.99	91.31	90.21	92.76	87.74	88.48	82.52	91.04	85.70	86.32	71.09	82.20	85.31	93.89
	Std	0.71	1.09	1.11	1.35	0.72	1.18	1.38	1.70	1.28	0.70	**0.62**	1.57	3.62	2.11	1.01	0.88
	Num	212.20	213.80	200.20	101.43	272.33	195.07	238.67	131.27	16.97	9.83	231.03	24.77	**3.90**	13.93	121.60	63.97
	Time	7.53	5.27	41.60	23.61	5.47	31.25	35.56	7.67	55.34	5.05	31.80	82.35	4.82	11.33	5.93	**4.81**
	Rank	**1**	5	3	6	8	4	10	9	14	7	12	11	16	15	13	2
*D*10	Acc	96.42	95.77	95.90	96.64	95.73	95.96	94.74	95.24	93.08	97.04	94.81	93.95	96.84	96.81	96.81	**98.04**
	Std	0.85	0.67	**0.43**	0.86	0.45	0.52	0.53	0.87	0.24	0.76	1.32	0.76	0.58	0.52	0.85	0.59
	Num	161.37	176.63	155.97	40.77	246.33	149.73	236.40	112.27	238.23	13.80	140.13	24.53	13.27	45.80	98.37	**9.73**
	Time	8.29	5.69	36.66	17.20	4.46	22.43	23.10	4.69	36.38	3.67	35.53	40.92	4.45	7.73	5.12	**3.83**
	Rank	7	10	9	6	11	8	14	12	16	2	13	15	3	4	4	**1**
*D*11	Acc	73.37	71.85	72.41	77.03	70.80	72.14	69.50	74.48	75.14	84.37	76.34	78.40	77.52	80.17	73.59	**86.35**
	Std	0.79	0.79	0.81	1.76	**0.45**	0.73	0.52	2.13	2.37	0.83	1.64	1.18	1.20	1.60	0.79	1.26
	Num	198.73	199.87	187.07	13.53	255.23	184.27	233.50	21.30	91.03	114.93	190.00	11.33	**5.33**	11.67	117.20	24.60
	Time	13.64	7.14	59.33	30.75	6.99	50.42	52.67	7.94	67.73	8.46	52.98	84.48	6.45	10.66	**5.21**	9.07
	Rank	11	14	12	6	15	13	16	9	8	2	7	4	5	3	10	**1**
*D*12	Acc	**100.00**	99.25	99.73	99.84	99.57	99.61	98.98	99.29	97.15	98.57	97.82	98.16	98.31	97.29	96.43	**100.00**
	Std	**0.00**	0.57	0.50	0.40	0.57	0.55	0.40	0.58	0.64	0.51	0.36	0.79	0.66	0.81	0.21	**0.00**
	Num	150.90	155.83	137.73	29.43	226.47	144.40	225.00	82.70	197.50	21.87	50.33	20.40	7.10	**6.33**	82.10	36.70
	Time	5.60	3.86	29.24	15.86	3.75	17.66	17.75	4.42	26.09	10.85	5.01	35.02	3.93	7.63	4.19	**3.54**
	Rank	**1**	8	4	3	6	5	9	7	15	10	13	12	11	14	16	**1**

**Fig. 3:**
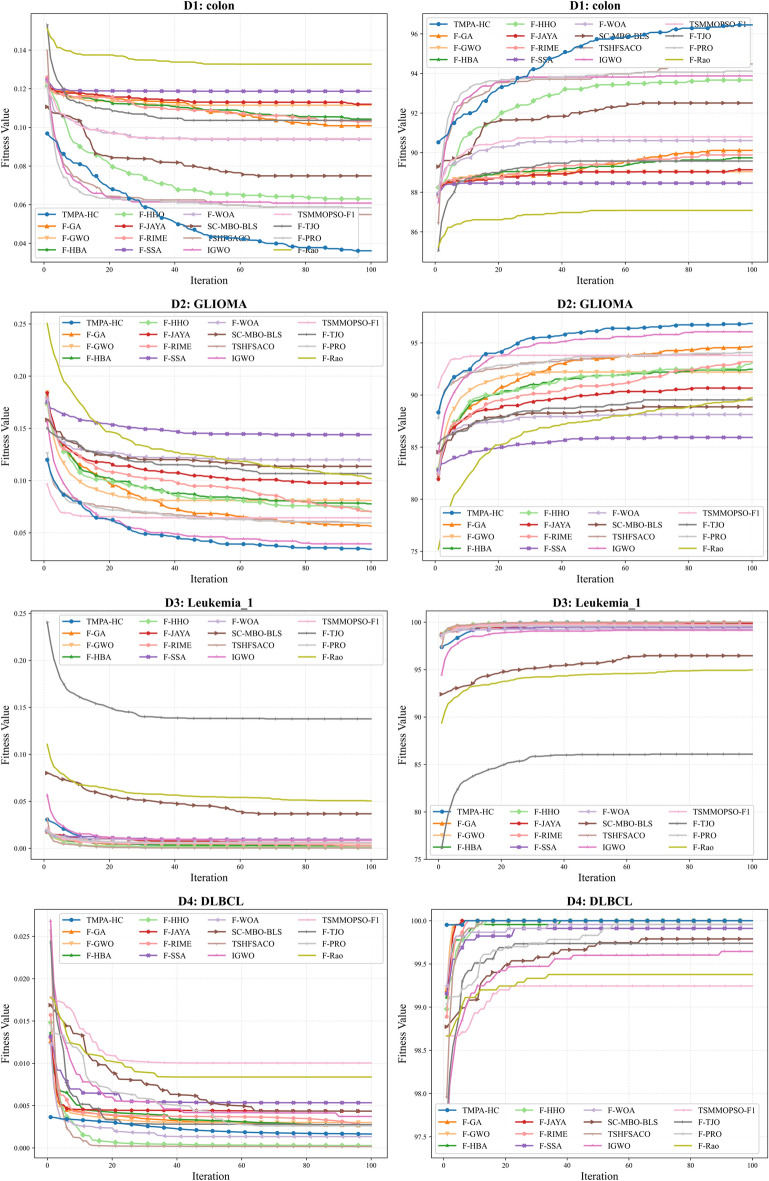
Comparison of convergence trends between TMPA-HC and other algorithms on datasets D1–D4.

As shown in Table 4 - Table 5, TMPA-HC achieved optimal accuracy in 9 out of 12 high-dimensional datasets, and also secured competitive second and third place rankings in the remaining 3 datasets. This fully demonstrates TMPA-HC’s excellent optimization capabilities on high-dimensional datasets. Furthermore, Fig. 3 - Fig. 5 show the convergence curves of each algorithm on the 12 high-dimensional datasets, including convergence curves for fitness and accuracy. We can observe that TMPA-HC’s convergence speed and convergence value are both among the highest.

Fig. 3 shows the convergence curves for the four datasets *D*1-*D*4. We can clearly observe that TMPA-HC significantly outperforms other algorithms in both convergence speed and convergence value on *D*1 and *D*2. Its accuracy on *D*1 and *D*2 reached $96.45\%$ and $96.87\%$ respectively, leading the second-place algorithm by $1.97\%$ and $0.80\%$, while using the shortest time, 4.15 seconds and 3.61 seconds respectively. Even though F-PRO exhibited rapid initial convergence and achieved good early-stage fitness values, it was overtaken by TMPA-HC around iteration 20, which reached a superior overall convergence. On *D*3 and *D*4, TMPA-HC’s fitness values did not fully reach the optimum, whereas its accuracy convergence achieved the maximum level. This is because our fitness function considers both classification accuracy and the size of the feature subset, compelling the algorithm to achieve high predictive performance while keeping the subset as compact as possible. The reason TMPA-HC’s fitness value didn’t reach the optimal level is that it selected more effective features, thus achieving $100\%$ accuracy on both datasets. Conversely, the F-HHO algorithm on *D*3, even with a lower convergence value in fitness than TMPA-HC, had lower accuracy. This decrease in accuracy is attributed to the removal of certain important features by the algorithm. Meanwhile, we found that on both *D*3 and *D*4 datasets, most algorithms using the Fisher filter achieved $100\%$ accuracy. This result clearly highlights the effectiveness of the Fisher filter in eliminating a large proportion of irrelevant or redundant features during the first stage, thereby providing the second-stage optimization process with a comparatively high-quality initial feature subset, as reflected by the early behaviour of the convergence curve. We also found that F-PRO selected the fewest 4.11 features when its accuracy on *D*4 was only $0.62\%$ lower than the first place.

**Fig. 4:**
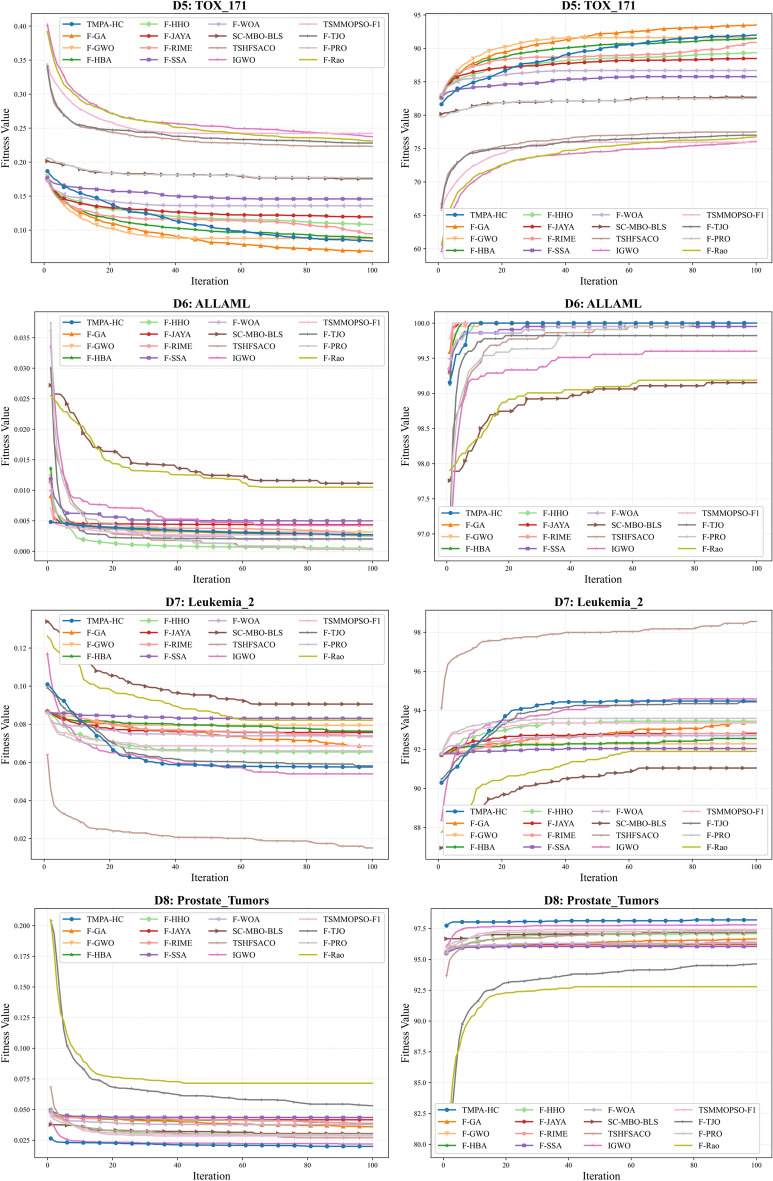
Comparison of convergence trends between TMPA-HC and other algorithms on datasets D5 - D8.

As shown in Fig. 4, TMPA-HC achieved first place on *D*6 and *D*8, and second and third place on *D*5 and *D*7, respectively. On *D*5, although TMPA-HC’s convergence speed was slower than algorithms like F-GWO and F-HBA in the early stages of iteration, it surpassed these algorithms after nearly 80 iterations, selecting a competitive feature subset and achieving a second-highest accuracy. Similar results to *D*3 and *D*4 were observed on dataset *D*6, where TMPA-HC and most algorithms using Fisher filters achieved $100\%$ accuracy. However, it’s worth noting that the F-SSA algorithm did not reach its optimal value. Furthermore, TMPA-HC still took the least time on *D*6. On *D*7, we can see that the TSHFSACO algorithm outperformed all algorithms by nearly 4 percentage points, achieving optimal convergence speed and convergence value. The TMPA-HC algorithm only excelled in runtime, ranking third with an accuracy of $94.48\%$. On *D*8, the TMPA-HC algorithm ranked first with the best convergence speed and an accuracy of $98.20\%$, surpassing the TSHFSACO algorithm by 1 percentage point. This is because the TSHFSACO algorithm filtered out many effective features, which led to a decrease in its accuracy.

**Fig. 5:**
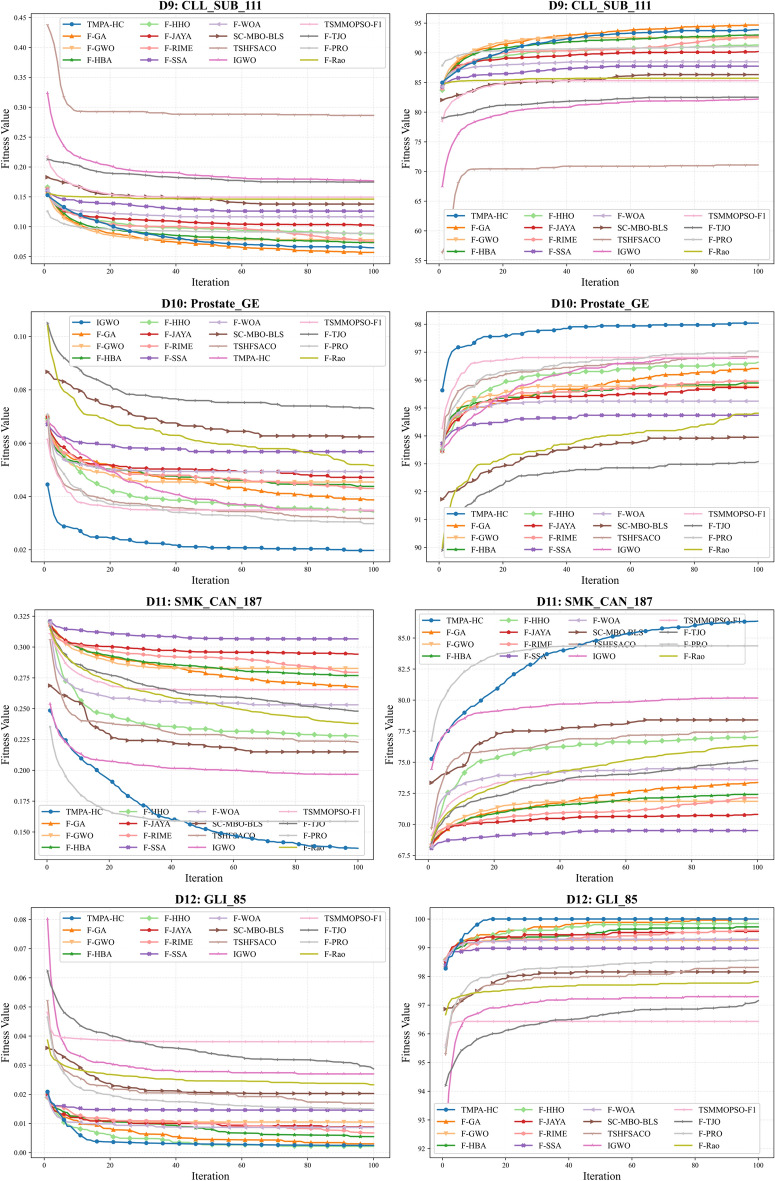
Comparison of convergence trends between TMPA-HC and other algorithms on datasets D9 - D12.

As shown in Fig. 5, TMPA-HC achieved a significant lead on the three highest-dimensional datasets. On the *D*9 dataset, although it didn’t reach the highest accuracy, it ranked second. However, F-GA selected an average of 212.20 features, while TMPA-HC selected only 63.97 features, less than one-third of F-GA’s selection. Yet, its accuracy was only $0.87\%$ lower than F-GA, indicating that the F-GA algorithm selected too many ineffective or redundant features. This explains why TMPA-HC took almost half the time of F-GA. On the *D*10 and *D*11 datasets, TMPA-HC achieved optimal convergence values for both fitness and accuracy. With an average of 9.73 and 24.60 features selected, it achieved $98.04\%$ and $86.35\%$ accuracy, respectively. On *D*10, it surpassed the second-place TSHFSACO algorithm by $1.20\%$ while selecting 3.54 fewer features and exhibiting extremely fast convergence. On *D*11, it surpassed the second-place algorithm by six percentage points, representing a significant lead. Meanwhile, we can see that although the TSHFSACO algorithm selects the fewest features (5.33), its accuracy is only $77.52\%$, indicating that it filters out many important or strongly correlated features. On *D*12, methods using the Fisher filter generally lead in initial values, but the TMPA-HC algorithm surpasses other similar algorithms in only about 10 iterations due to its excellent optimization ability, achieving a lead. After 20 iterations, it converges to the optimal value, reaching $100\%$ accuracy. The F-GA algorithm, however, only reaches the same level after nearly 90 iterations towards the end of the iteration process.

To rigorously evaluate performance differences between TMPA-HC and the benchmark algorithms, statistical analyses were conducted using the Friedman procedure in combination with the Wilcoxon signed-rank test and Holm’s adjustment for multiple comparisons. Table 6 presents the average ranks, final standings, and $95\%$ confidence intervals for all algorithms across the experiments. As indicated, TMPA-HC attains the top position overall, with the lowest mean rank of 2.417 and the highest final ranking, corresponding to a $95\%$ confidence interval of [93.53, 98.82]. In comparison, F-HHO and F-GA rank second and third, with average ranks of 6.125 and 6.208, respectively, while the other methods show notably higher mean ranks. These findings confirm that TMPA-HC holds a statistically significant advantage across the test set, and the Friedman-based analysis further highlights substantial performance differences among the evaluated algorithms.

**Table 6:** Friedman ranking analysis of algorithms with 95% confidence intervals.

Algorithm	Mean Rank	Overall Rank	95% CI
TMPA-HC	2.417	1	$\left[ 93.53, 98.82 \right]$
F-HHO	6.125	2	$\left[ 89.85, 98.29 \right]$
F-GA	6.208	3	$\left[ 89.49, 98.87 \right]$
F-PRO	6.250	4	$\left[ 90.63, 98.06 \right]$
TSHFSACO	7.417	5	$\left[ 85.60, 98.81 \right]$
F-HBA	7.458	6	$\left[ 88.83, 98.54 \right]$
TSMMOPSO-F1	7.625	7	$\left[ 86.36, 98.06 \right]$
F-GWO	8.542	8.5	$\left[ 88.46, 98.36 \right]$
F-JAYA	8.542	8.5	$\left[ 87.79, 98.24 \right]$
IGWO	8.708	10	$\left[ 87.34, 97.86 \right]$
F-RIME	8.917	11	$\left[ 87.91, 98.06 \right]$
SC-MBO-BLS	9.000	12	$\left[ 88.30, 97.28 \right]$
F-WOA	10.083	13	$\left[ 87.78, 97.04 \right]$
F-SSA	12.458	14	$\left[ 86.16, 97.28 \right]$
F-TJO	12.667	15	$\left[ 84.63, 95.15 \right]$
F-Rao	13.583	16	$\left[ 85.55, 95.53 \right]$

To investigate whether TMPA-HC shows statistically significant differences relative to the benchmark algorithms, pairwise comparisons were conducted using the Wilcoxon signed-rank test, with Holm’s procedure applied to correct for multiple comparisons. Pairwise comparison results, presented in Table 7, indicate that for algorithms such as F-GWO, F-HBA, F-HHO, F-JAYA, F-RIME, F-SSA, F-WOA, F-TJO, F-PRO, F-Rao, SC-MBO-BLS, and IGWO, all Holm-adjusted p-values fall below 0.05, resulting in the rejection of the null hypotheses and confirming a significant performance advantage for TMPA-HC. Conversely, comparisons with F-GA, TSHFSACO, and TSMMOPSO-F1 show that although TMPA-HC achieves superior average performance, the Holm-corrected p-values exceed 0.05, leading to acceptance of the null hypotheses and indicating no statistically significant difference. Overall, these Wilcoxon–Holm test results corroborate the Friedman ranking analysis, demonstrating that TMPA-HC maintains stable and significant superiority over most competing algorithms.

**Table 7:** Results of Wilcoxon signed-rank tests with holm’s correction for TMPA-HC.

Comparison	R+	R-	Stat	p-value	Corrected p	Hypothesis
F-GA	63.0	15.0	15.0	0.0593	0.1187	*Accepted*
F-GWO	75.0	3.0	3.0	0.0047	0.0260	*Rejected*
F-HBA	76.5	1.5	1.5	0.0033	0.0260	*Rejected*
F-HHO	76.5	1.5	1.5	0.0033	0.0260	*Rejected*
F-JAYA	75.0	3.0	3.0	0.0047	0.0260	*Rejected*
F-RIME	77.5	0.5	0.5	0.0025	0.0253	*Rejected*
F-SSA	78.0	0.0	0.0	0.0005	0.0059	*Rejected*
F-WOA	77.5	0.5	0.5	0.0025	0.0253	*Rejected*
F-TJO	78.0	0.0	0.0	0.0005	0.0073	*Rejected*
F-PRO	77.5	0.5	0.5	0.0025	0.0278	*Rejected*
F-Rao	78.0	0.0	0.0	0.0005	0.0073	*Rejected*
SC-MBO-BLS	76.5	1.5	1.5	0.0033	0.0260	*Rejected*
TSHFSACO	62.5	15.5	15.5	0.0653	0.1187	*Accepted*
IGWO	77.0	1.0	1.0	0.0010	0.0107	*Rejected*
TSMMOPSO-F1	68.0	10.0	10.0	0.0227	0.0681	*Accepted*

In conclusion, the outcomes of the statistical analyses strongly confirm the overall performance superiority of TMPA-HC across the considered test problems. Findings from the Friedman ranking and Wilcoxon–Holm tests collectively show that TMPA-HC attains statistically significant improvements over most competing algorithms, indicating that the proposed enhancement strategies effectively and consistently enhance both search efficiency and solution quality.

### Classifier comparison study

SVM, due to its strong margin maximization principle and solid theoretical foundation in structural risk minimization, has been widely employed in high-dimensional small-sample classification tasks. In contrast, XGBoost (XGB), a representative ensemble learning method based on gradient boosting decision trees, has demonstrated excellent predictive performance in many large-scale machine learning scenarios. To investigate whether the effectiveness of TMPA-HC depends on a specific classifier choice, a comparative study between SVM and XGBoost was conducted.

The average classification accuracies over 30 independent runs under the 5-fold cross-validation protocol are summarized in Table 8. It can be observed that SVM achieves higher accuracy on 9 out of the 12 datasets, while both classifiers attain identical performance on *D*3 and *D*4. XGBoost outperforms SVM only on *D*5, where the margin is relatively small ($0.85\%$). From an overall statistical perspective, the mean classification accuracy of SVM across all datasets is $96.36\%$, whereas XGBoost achieves $95.69\%$, resulting in an average improvement of approximately 0.67 percentage points. The performance gap between the two classifiers is generally limited within $1--2\%$ for most datasets. For example, on *D*6 and *D*12, SVM improves upon XGBoost by $0.26\%$ and $0.15\%$, respectively, indicating that both classifiers perform nearly identically when the selected feature subset is highly discriminative. On moderately challenging datasets such as *D*7, *D*9, and *D*11, the improvement ranges from $0.83\%$ to $1.51\%$, suggesting that SVM exhibits slightly stronger stability under more difficult classification conditions.

The superior performance of SVM in this study can be attributed to the intrinsic characteristics of microarray gene expression datasets, where the number of samples is extremely limited compared to the feature dimension. Under such high-dimensional small-sample conditions, margin-based classifiers tend to provide better generalization capability, whereas tree-based ensemble methods may introduce higher variance due to insufficient data for reliable node splitting. Nevertheless, the competitive performance of XGBoost across most datasets further demonstrates that the feature subsets selected by TMPA-HC retain strong discriminative information and are not overly tailored to a specific classifier.

**Table 8:** Performance comparison of different classifiers based on average accuracy across 12 datasets.

Dataset	XGBoost	SVM
*D*1	95.62	**96.45**
*D*2	95.13	**96.87**
*D*3	**100.00**	**100.00**
*D*4	**100.00**	**100.00**
*D*5	**92.84**	91.99
*D*6	99.74	**100.00**
*D*7	93.65	**94.48**
*D*8	97.41	**98.20**
*D*9	92.38	**93.89**
*D*10	96.77	**98.04**
*D*11	84.92	**86.35**
*D*12	99.85	**100.00**

In summary, although SVM demonstrates slightly superior average performance, the relatively small differences between the two classifiers confirm that TMPA-HC produces stable and transferable feature subsets. These findings justify the selection of SVM as the default classifier while also validating the robustness of the proposed feature selection framework across different classification paradigms.

### Parameter sensitivity analysis

**Fig. 6:**
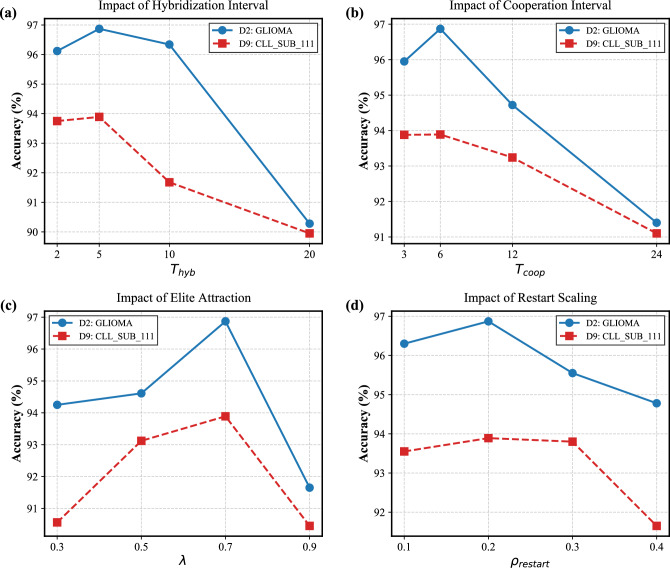
Parameter sensitivity analysis of TMPA-HC on datasets *D*2 and *D*9 with respect to (**a**) Hybridization interval $T_{hyb}$, (**b**) Cooperation interval $T_{coop}$, (**c**) Elite attraction strength $\lambda$, and (**d**) Restart scaling factor $\rho _{restart}.$.

To empirically validate the robustness of TMPA-HC and verify the rationality of the default parameter settings listed in Table 1, we conducted a sensitivity analysis on two representative datasets: *GLIOMA* (*D*2, representing high-dimensional data with small sample size) and *CLL_SUB_111* (*D*9, representing ultra-high-dimensional data). We systematically varied four critical parameters: the hybridization interval $T_{hyb}$, the cooperation interval $T_{coop}$, the elite attraction strength $\lambda$, and the restart scaling factor $\rho _{restart}$. The experimental results are illustrated in Fig. 6.

As shown in Fig. 6 (a), the hybridization interval $T_{hyb}$ plays a crucial role in population diversity. Both datasets achieve optimal accuracy when $T_{hyb}$ is set to 5. While a frequent exchange ($T_{hyb}=2$) yields acceptable results, a significant performance degradation is observed when the interval becomes too large ($T_{hyb}=20$). For instance, on dataset *D*2, accuracy drops sharply from 96.87% (at $T_{hyb}=5$) to 90.28% (at $T_{hyb}=20$). This suggests that infrequent information sharing hinders the timely transfer of beneficial features, causing subpopulations to stagnate. Similarly, regarding the cooperation interval $T_{coop}$ in Fig. 6 (b), the algorithm exhibits a clear peak at $T_{coop}=6$. Unlike the hybridization parameter, $T_{coop}$ shows higher sensitivity; increasing the interval to 12 or 24 leads to a noticeable decline in accuracy for both datasets (e.g., *D*2 drops to 91.40% at $T_{coop}=24$). This indicates that the cooperative mechanism–designed to assist the worst-performing individuals–must be activated with moderate frequency to be effective. The default settings ($T_{hyb}=5, T_{coop}=6$) are therefore confirmed as the optimal configuration for balancing exploration and exploitation.

Figure 6 (c) illustrates the effect of the elite attraction parameter $\lambda$. The performance shows a distinct inverted-U shape. Accuracy improves significantly as $\lambda$ increases from 0.3 to 0.7, highlighting the importance of elite guidance in high-dimensional spaces. However, a critical observation is the sharp decline when $\lambda$ reaches 0.9 (e.g., *D*9 drops to 90.45%). This substantial deterioration indicates that excessive attraction to the current best solution causes the population to collapse into local optima prematurely. Thus, $\lambda =0.7$ provides the necessary trade-off. Finally, Fig. 6 (d) shows the sensitivity to the restart scaling factor $\rho _{restart}$. The performance is relatively stable between 0.1 and 0.2, peaking at $\rho _{restart}=0.2$. However, when the perturbation becomes too aggressive ($\rho _{restart}=0.4$), the accuracy decreases (e.g., *D*9 drops to 91.65%), as the search trajectory is disrupted before fine-tuning can occur.

In summary, the sensitivity analysis confirms that the proposed TMPA-HC achieves its best performance with the default parameters listed in Table 1. While the algorithm is robust to small variations, extreme parameter values (particularly large intervals or excessive elite pressure) can negatively impact feature selection accuracy, justifying our specific parameter choices.

### Ablation study

To assess the contribution of individual components in TMPA-HC, an ablation experiment was performed by selectively removing each strategy. As reported in Table 9, the full TMPA-HC consistently achieves the highest classification accuracy across all datasets. These results suggest that the coordinated combination of the various mechanisms contributes to the algorithm’s performance, with no single component being superfluous.

**Table 9:** Ablation study results of TMPA-HC in terms of classification accuracy (%).

Dataset	TMPA-HC	TMPA-HC–RS	TMPA-HC–REO	TMPA-HC–LF	TMPA-HC–CO	TMPA-HC–AD	TMPA-HC–2SP	TMPA-HC–4SP	MPA-HC
*D*1	96.45	95.98	95.72	94.83	93.94	92.31	93.02	94.36	89.74
*D*2	96.87	96.31	96.05	95.02	94.10	92.88	93.41	94.92	90.83
*D*3	100.00	99.87	99.74	99.42	99.18	98.65	98.74	99.21	96.38
*D*4	100.00	99.91	99.85	99.60	99.32	98.94	98.86	99.32	96.74
*D*5	91.99	91.28	90.74	89.36	87.92	86.15	85.94	87.31	80.65
*D*6	100.00	99.92	99.86	99.63	99.41	98.97	98.92	99.35	96.88
*D*7	94.48	93.87	93.46	92.71	91.95	90.38	90.76	92.03	86.14
*D*8	98.20	97.81	97.46	96.78	95.92	94.67	94.81	96.12	90.73
*D*9	93.89	93.14	92.58	91.26	89.83	87.96	88.97	90.52	83.48
*D*10	98.04	97.62	97.28	96.41	95.56	94.38	95.02	96.37	91.85
*D*11	86.35	85.62	84.97	83.54	81.76	79.88	80.14	82.09	74.63
*D*12	100.00	99.89	99.76	99.43	99.11	98.72	98.69	99.17	95.92

When individual strategies are removed, varying degrees of performance degradation can be observed. Disabling the adaptive control mechanism (TMPA-HC–AD) leads to the most significant accuracy loss on almost all datasets, especially on more challenging ones, demonstrating that success-rate–driven parameter adaptation is essential for maintaining a proper exploration–exploitation balance. Removing cross-subpopulation cooperation (TMPA-HC–CO) also causes a clear decline, as the absence of information exchange among heterogeneous subpopulations limits global search capability. The elimination of the Lévy-flight mechanism (TMPA-HC–LF) results in moderate but consistent performance drops, suggesting that long-range perturbations are effective in escaping local optima. In contrast, removing subpopulation reorganization (TMPA-HC–REO) and restart with local intensification (TMPA-HC–RS) leads to relatively smaller degradations, indicating that these strategies mainly enhance search stability and convergence robustness rather than directly driving global optimization.

More importantly, structural validation experiments further clarify the rationality of the tri-subpopulation architecture. When reducing the framework to two subpopulations (TMPA-HC–2SP), noticeable deterioration occurs across all datasets. On *D*5, *D*9, and *D*11, the reductions relative to TMPA-HC reach 6.05%, 4.92%, and 6.21%, respectively. The absence of the balance subgroup disrupts the intended coordination between exploration and exploitation, leading to insufficient adaptability in complex search landscapes. In contrast, introducing an additional fourth subgroup (TMPA-HC–4SP) partially mitigates this issue compared to 2SP but still fails to match the full TMPA-HC. Although diversity is increased, excessive subdivision disperses search effort and weakens efficient information fusion. The empirical results demonstrate that the three-subpopulation configuration provides a more appropriate trade-off between diversity maintenance and convergence efficiency.

Furthermore, removing the Fisher-based filtering stage (MPA-HC) yields the most dramatic overall degradation. On high-dimensional datasets such as *D*5, *D*9, and *D*11, accuracy decreases by 11.34%, 10.41%, and 11.72%, respectively. Even on easier datasets like *D*3 and *D*4, noticeable drops remain. This phenomenon clearly indicates that preliminary Fisher-score filtering substantially reduces irrelevant and redundant features before evolutionary optimization. Without this dimensionality reduction step, the wrapper search operates directly in a vast noisy feature space, significantly enlarging the search domain and diminishing optimization efficiency.

Overall, the ablation results confirm that the superior performance of TMPA-HC is not attributed to a single technique, but rather to the synergistic combination of adaptive control, cooperative search, enhanced exploration, robustness-oriented mechanisms, three-subpopulation configuration, and Fisher-based filtering. The combined integration of these components allows TMPA-HC to deliver consistent and competitive performance across a variety of high-dimensional feature selection tasks.

### Evolutionary search dynamics and mechanism analysis

**Fig. 7:**
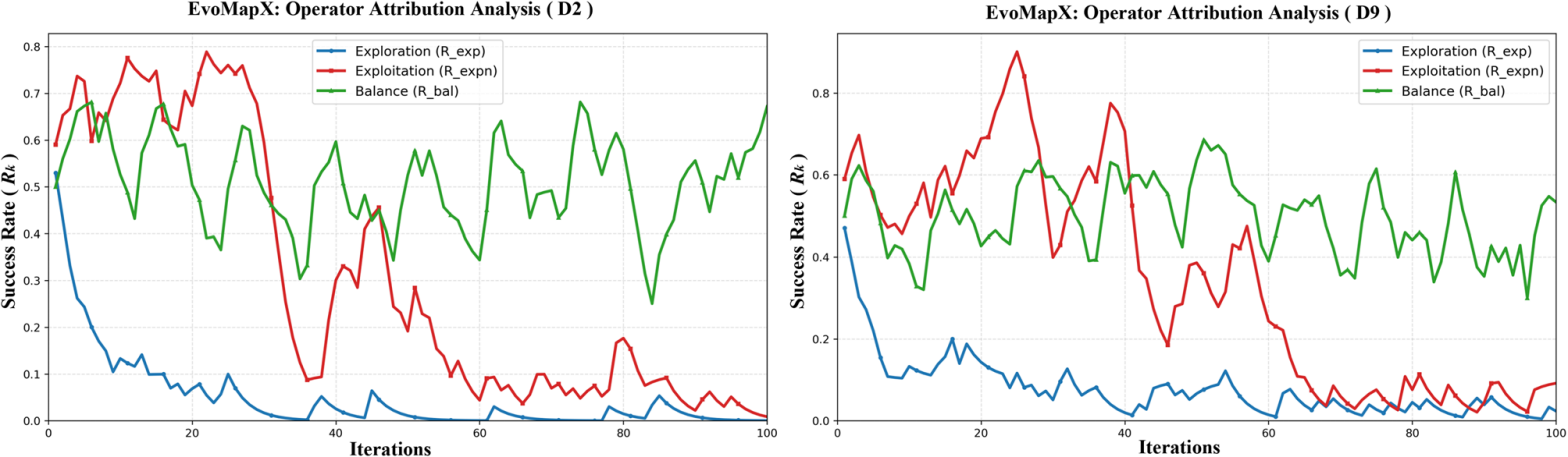
Evolutionary dynamics of subpopulation success rates ($R_k$) on (**a**) GLIOMA (D2) and (**b**) CLL_SUB_111 (D9). The curves illustrate the distinct functional roles of exploration, exploitation, and balance strategies, revealing the heterogeneous search behavior during the optimization process.

To enhance the interpretability and provide deeper insights into the internal search behavior of TMPA-HC, we conducted an Operator Attribution Analysis on two representative datasets: D2 and D9. The success-rate curves correspond to the operator contribution dynamics in EvoMapX, reflecting the relative effectiveness of each subpopulation during different evolutionary stages. Figure 7 visualizes the dynamic evolution of the $R_k$ for the three heterogeneous subpopulations, revealing three distinct evolutionary phases that perfectly align with the proposed heterogeneous design. Initially, the Exploration subpopulation (Blue Line) exhibits a high success rate only in the early phase (iterations 1–15) and decays rapidly thereafter. This confirms that the Lévy-flight-based operators effectively identify promising basins of attraction early on, avoiding inefficient random search in later stages when the population has converged. Subsequently, the Exploitation subpopulation (Red Line) dominates the early-to-mid stage. Its success rate peaks rapidly around iteration 10 for D2, whereas for the higher-dimensional D9, the peak is delayed to iterations 20–40. This delay logically reflects the increased difficulty of locating optima in ultra-high-dimensional spaces, proving the algorithm’s adaptability. Crucially, the Balance subpopulation (Green Line) maintains a robust success rate (fluctuating between 0.4 and 0.6) throughout the entire process, especially in the late stages (iterations 60–100) where the other two subpopulations stagnate with success rates approaching zero.

This analysis empirically validates the results of the Ablation Study. For instance, the performance drop observed in the variant without the Balance group (TMPA-HC-2SP) can now be explicitly attributed to the lack of effective search operators in the late evolutionary stages. The visual evidence confirms that the Balance subpopulation acts as the primary engine for continuous improvement when pure exploration or exploitation strategies become ineffective, thereby preventing premature convergence. By explicitly assigning distinct roles to heterogeneous subpopulations, TMPA-HC achieves a synergistic balance, ensuring sustained optimization capability across different phases of the search process.

## Conclusions

This study proposes TMPA-HC, a two-stage heterogeneous multi-population algorithm with cooperative search for high-dimensional feature selection. By integrating an initial Fisher-score-based filtering stage with a heterogeneous multi-subpopulation evolutionary framework, TMPA-HC effectively addresses the challenges of feature redundancy and high computational cost commonly encountered in traditional evolutionary feature selection methods. The incorporation of adaptive control, cross-subpopulation cooperation, and robustness-oriented strategies enables the algorithm to maintain population diversity while achieving stable and efficient convergence. Extensive experiments on continuous optimization benchmarks and high-dimensional biomedical datasets demonstrate that TMPA-HC consistently achieves superior or comparable performance relative to state-of-the-art algorithms in terms of classification accuracy, convergence behavior, and robustness. Furthermore, the ablation study and operator attribution analysis confirm that the superior performance arises from the synergistic interaction of its core mechanisms rather than reliance on a single strategy.

Despite these promising results, several limitations should be acknowledged to provide a balanced view of the algorithm’s applicability. First, the multi-subpopulation structure and adaptive mechanisms introduce additional computational overhead compared to simpler frameworks. Consequently, for small-scale or low-dimensional problems where the search landscape is relatively smooth, the performance gain may not justify the increased algorithmic complexity, and lightweight single-population methods might be more efficient. Second, while the Fisher-based preprocessing significantly reduces dimensionality, its reliance on linear criteria risks discarding weak but potentially complementary features that could be crucial for highly non-linear classification problems. Finally, like most population-based metaheuristics, the algorithm involves several control parameters, and performance sensitivity to parameter calibration remains a consideration when applying the method to domains substantially different from those evaluated in this study.

Future work will focus on addressing these limitations and extending the framework’s capabilities. Specifically, we plan to investigate adaptive mechanisms that can automatically adjust the algorithmic complexity based on the problem scale, thereby improving efficiency on lower-dimensional tasks. Additionally, exploring non-linear filtering techniques or integrating embedded feature selection strategies could help capture more complex feature interactions. We also aim to extend TMPA-HC to multi-objective optimization frameworks to simultaneously balance feature subset size and classification accuracy in larger-scale real-world applications.

## Data Availability

The datasets used in this paper can be accessed via the following link (UCI Machine Learning Repository): https://archive.ics.uci.edu/.
